# Impact of Replacing Smear Microscopy with Xpert MTB/RIF for Diagnosing Tuberculosis in Brazil: A Stepped-Wedge Cluster-Randomized Trial

**DOI:** 10.1371/journal.pmed.1001766

**Published:** 2014-12-09

**Authors:** Betina Durovni, Valeria Saraceni, Susan van den Hof, Anete Trajman, Marcelo Cordeiro-Santos, Solange Cavalcante, Alexandre Menezes, Frank Cobelens

**Affiliations:** 1 Rio de Janeiro Municipal Health Secretariat, Rio de Janeiro, Brazil; 2 Programa de Pós-graduação em Clínica Médica, Rio de Janeiro Federal University, Rio de Janeiro, Brazil; 3 Programa de Pós-graduação em Doenças Infecciosas, Tropical Medicine Foundation Dr. Heitor Vieira Dourado, Manaus, Brazil; 4 KNCV Tuberculosis Foundation, The Hague, The Netherlands; 5 Department of Global Health, Academic Medical Center and Amsterdam Institute for Global Health and Development, Amsterdam, The Netherlands; 6 Montreal Chest Institute, McGill University, Montreal, Canada; 7 Amazonas State University, Manaus, Brazil; 8 Oswaldo Cruz Foundation, Instituto de Pesquisa Evandro Chagas, Rio de Janeiro, Brazil; 9 Global Health Strategies, Rio de Janeiro, Brazil; Harvard School of Public Health, United States of America

## Abstract

**Background:**

Abundant evidence on Xpert MTB/RIF accuracy for diagnosing tuberculosis (TB) and rifampicin resistance has been produced, yet there are few data on the population benefit of its programmatic use. We assessed whether the implementation of Xpert MTB/RIF in routine conditions would (1) increase the notification rate of laboratory-confirmed pulmonary TB to the national notification system and (2) reduce the time to TB treatment initiation (primary endpoints).

**Methods and Findings:**

We conducted a stepped-wedge cluster-randomized trial from 4 February to 4 October 2012 in 14 primary care laboratories in two Brazilian cities. Diagnostic specimens were included for 11,705 baseline (smear microscopy) and 12,522 intervention (Xpert MTB/RIF) patients presumed to have TB. Single-sputum-sample Xpert MTB/RIF replaced two-sputum-sample smear microscopy for routine diagnosis of pulmonary TB. In total, 1,137 (9.7%) tests in the baseline arm and 1,777 (14.2%) in the intervention arm were positive (*p*<0.001), resulting in an increased bacteriologically confirmed notification rate of 59% (95% CI = 31%, 88%). However, the overall notification rate did not increase (15%, 95% CI = −6%, 37%), and we observed no change in the notification rate for those without a test result (−3%, 95% CI = −37%, 30%). Median time to treatment decreased from 11.4 d (interquartile range [IQR] = 8.5–14.5) to 8.1 d (IQR = 5.4–9.3) (*p* = 0.04), although not among confirmed cases (median 7.5 [IQR = 4.9–10.0] versus 7.3 [IQR = 3.4–9.0], *p* = 0.51). Prevalence of rifampicin resistance detected by Xpert was 3.3% (95% CI = 2.4%, 4.3%) among new patients and 7.4% (95% CI = 4.3%, 11.7%) among retreatment patients, with a 98% (95% CI = 87%, 99%) positive predictive value compared to phenotypic drug susceptibility testing. Missing data in the information systems may have biased our primary endpoints. However, sensitivity analyses assessing the effects of missing data did not affect our results.

**Conclusions:**

Replacing smear microscopy with Xpert MTB/RIF in Brazil increased confirmation of pulmonary TB. An additional benefit was the accurate detection of rifampicin resistance. However, no increase on overall notification rates was observed, possibly because of high rates of empirical TB treatment.

**Trial registration:**

ClinicalTrials.gov NCT01363765

*Please see later in the article for the Editors' Summary*

## Abstract 2

Betina Durovni and colleagues evaluated whether implementation of Xpert MTB/RIF increased the notification rate of laboratory-confirmed pulmonary tuberculosis and reduced the time to tuberculosis treatment initiation in 14 Brazilian primary care laboratories.

*Please see later in the article for the Editors' Summary*

## Editors' Summary

**Background:**

Tuberculosis—a contagious bacterial disease that usually infects the lungs—is a global public health problem. Each year, about 8.6 million people develop active tuberculosis and at least 1.3 million people die from the disease, mainly in resource-limited countries. *Mycobacterium tuberculosis*, the bacterium that causes tuberculosis, is spread in airborne droplets when people with active disease cough or sneeze. The characteristic symptoms of tuberculosis include cough, weight loss, and night sweats. Diagnostic tests for tuberculosis include sputum smear microscopy (microscopic analysis of mucus coughed up from the lungs), the growth (culture) of *M. tuberculosis* from sputum samples, and molecular tests (for example, the Xpert MTB/RIF test) that rapidly and accurately detect *M. tuberculosis* in sputum and determine its antibiotic resistance. Tuberculosis can be cured by taking several antibiotics daily for at least six months, although the emergence of multidrug-resistant tuberculosis is making the disease increasingly hard to treat.

**Why Was This Study Done?:**

Quick, accurate diagnosis of active tuberculosis is essential to reduce the global tuberculosis burden, but in most high-burden settings diagnosis relies on sputum smear analysis, which fails to identify many infected people. Mycobacterial culture correctly identifies more infected people but is slow, costly, and rarely available in resource-limited settings. In late 2010, therefore, the World Health Organization recommended the routine use of the Xpert MTB/RIF assay (Xpert) for tuberculosis diagnosis, and several resource-limited countries are currently scaling up the use of Xpert in their national tuberculosis control programs. However, although Xpert works well in ideal conditions, little is known about its performance in routine (real-life) settings. In this pragmatic stepped-wedge cluster-randomized trial, the researchers assess the impact of replacing smear microscopy with Xpert for the diagnosis of tuberculosis in Brazil, an upper-middle-income country with a high tuberculosis burden. A pragmatic trial asks whether an intervention works under real-life conditions; a stepped-wedge cluster-randomized trial sequentially and randomly rolls out an intervention to groups (clusters) of people.

**What Did the Researchers Do and Find?:**

The researchers randomly assigned 14 tuberculosis diagnosis laboratories in two cities to switch at different times from smear microscopy to Xpert for tuberculosis diagnosis. Specifically, at the start of the eight-month trial, all the laboratories used smear microscopy for tuberculosis diagnosis. At the end of each month, two laboratories switched to using Xpert, so that in the final month of the trial, all the laboratories were using Xpert. During the trial, 11,705 samples from patients with symptoms consistent with tuberculosis were examined using smear microscopy (baseline arm), and 12,522 samples were examined using Xpert (intervention arm). The researchers obtained the results of these tests from a database of all the diagnostic tests ordered in the Brazilian public laboratory system, and they obtained data on tuberculosis notifications during the trial period from the national notification system. In total, 9.7% and 14.2% of the tests in the baseline and intervention arm, respectively, were positive, and the laboratory-confirmed tuberculosis notification rate was 1.59 times higher in the Xpert arm than in the smear microscopy arm. However, the overall notification rate (which included people who began treatment on the basis of symptoms alone) did not increase during the trial. The time to treatment (the time between the laboratory test date and the notification date, when treatment usually starts in Brazil) was about 11 days and eight days in the smear microscopy and Xpert arms, respectively.

**What Do These Findings Mean?:**

The findings indicate that, in a setting where laboratory diagnosis for tuberculosis was largely restricted to sputum smear examination, the implementation of Xpert increased the rates of laboratory-confirmed pulmonary (lung) tuberculosis notifications and reduced the time to treatment initiation, two endpoints of public health relevance. However, implementation of Xpert did not increase the overall notification rate of pulmonary tuberculosis (probably because of the high rate of empiric tuberculosis treatment in Brazil), although it did facilitate accurate and rapid detection of rifampicin resistance. The accuracy of these findings may be limited by certain aspects of the trial design, and further studies are needed to evaluate the possible effects of Xpert beyond diagnosis and the time to treatment initiation. Nevertheless, these findings suggest that replacing smear microscopy with Xpert has the potential to increase the confirmation (but not detection) of pulmonary tuberculosis and to reduce the time to treatment initiation at the population level in Brazil and other resource-limited countries.

**Additional Information:**

Please access these websites via the online version of this summary at http://dx.doi.org/10.1371/journal.pmed.1001766.

- The World Health Organization (WHO) provides information (in several languages) on tuberculosis, on tuberculosis diagnostics, and on the rollout of Xpert; further information about WHO's endorsement of Xpert is included in a Strategic and Technical Advisory Group for Tuberculosis report; the “Global Tuberculosis Report 2013” provides information about tuberculosis around the world, including Brazil
- The Stop TB Partnership is working towards tuberculosis elimination and provides patient stories about tuberculosis (in English and Spanish); the Tuberculosis Vaccine Initiative (a not-for-profit organization) also provides personal stories about tuberculosis
- The US Centers for Disease Control and Prevention provides information about tuberculosis and its diagnosis (in English and Spanish)
- The US National Institute of Allergy and Infectious Diseases also has detailed information on all aspects of tuberculosis
- More information about this trial is available

## Introduction

The battle against tuberculosis (TB), a leading cause of death worldwide [1], has been hampered by a lack of accurate and rapid diagnostic tests, including those for drug resistance. The automated real-time PCR-based Xpert MTB/RIF assay (Xpert; Cepheid, Sunnyvale, California, US) can detect in 2 h the presence of a *Mycobacterium tuberculosis*–specific sequence of the *rpoB* gene as well as mutations in this gene responsible for most cases of phenotypic rifampicin resistance [2]. Xpert has proved to be feasible [3],[4], accurate [5], and cost-effective [6]–[8] under field conditions in different settings, including at point of care in peripheral clinics. Since the World Health Organization (WHO) endorsed the use of Xpert in populations with high rates of drug-resistant TB and HIV co-infection [9] in 2010, more than 85 peer-reviewed papers have reported the assay's accuracy for different specimens and populations [10].

However, the true clinical and public health performance of diagnostic tests is influenced by the treatment decisions made based on test results, and on delays in processing samples and reporting results [11]. For scaling up new diagnostics, decision-makers need pragmatic randomized controlled trials with patient-relevant endpoints, such as time to treatment initiation and treatment outcomes [12],[13]. A recent randomized controlled trial in sub-Saharan Africa [4] was the first to demonstrate that despite a higher proportion of TB confirmation for Xpert than for smear microscopy (83% versus 50%), overall TB detection did not increase, because of the high rates of empirical treatment. In addition, Xpert diagnosis did not result in decreased morbidity at 2 and 6 mo of treatment. Studies conducted in different populations (populations with high HIV co-infection rates [14] and hospitalized patients [15]) also failed to show improvement in clinical outcomes, despite the reduced time to TB diagnosis with Xpert [14],[15]. In both cases, rates of empirical treatment were also very high.

Moreover, substantial controversies remain about where Xpert capability should be located (peripheral clinics versus centralized laboratories), Xpert's role in increasing detection of drug-susceptible as well as multidrug-resistant (MDR) TB, and the optimal management of Xpert rifampicin-resistant cases before confirmatory phenotypic drug susceptibility testing (DST) results are available [10]. Also, the benefits of implementation of Xpert in routine medical care remain to be established. Despite the limited available evidence of the programmatic benefits of the adoption of the assay, by September 2013, 95 out of the 145 countries eligible for concessional prices had procured cartridges for the public sector [16].

In the context of a pilot rollout project in Brazil, we conducted a pragmatic trial to evaluate the effect of replacing two-sample smear examinations by one-sample Xpert on pulmonary TB notification to the national notification system and time to treatment initiation in routine public health practice. The trial's design, analysis, and reporting adhered to the principles of the CONSORT statement for pragmatic trials [17].

## Methods

### Ethics

The study was approved by the Brazil National Ethics Commission (CONEP #494/2011), the Rio de Janeiro Municipal Health Department Review Board (CEP SMS #236/11), and the Tropical Medicine Foundation of Amazonas Review Board (CEP FMT/HVD, 24 November 2011). The need for informed consent was waived by the ethical boards because this was a pilot implementation of a diagnostic test in routine practice, and only routine reporting data were used for the analysis.

### Study Setting and Participants

With 82,775 TB patients notified to Brazil's national notification system (Sistema de Informação de Agravos de Notificação [SINAN]) in 2012, Brazil is one of the 22 high-TB-burden countries [18]. Sputum smear examination (stained for acid-fast bacilli) is the mainstay of pulmonary TB diagnosis, with mycobacterial culture and DST recommended for specific sub-populations only, in particular for previously treated patients [19]. An estimated 26% of new patients start treatment on clinical/radiological grounds, without bacteriological confirmation, and in over 70% of retreatment patients no culture or DST is performed [20]. The Brazilian National TB Program recommends empirical treatment while awaiting culture results if, despite a course of broad antibiotics, symptoms persist and there is a high clinical suspicion despite negative smear results (Figure S1) [19]. Rates of co-infection with HIV (9.7%) and of rifampicin resistance (<2% in 2010; Draurio Barreira, Director of the Brazilian National TB Program, personal communication) are relatively low.

The study was conducted in the cities of Manaus and Rio de Janeiro, which notified 1,315 and 4,959 new pulmonary TB cases [21], respectively, in 2011. In Rio de Janeiro (2010 population: 6,320,446) [22], Xpert was introduced in all 11 public primary care laboratories. In Manaus (2010 population: 1,802,014) [22], Xpert was introduced in three public laboratories, including an HIV referral hospital and a TB referral center. These laboratories cover 70% of TB diagnoses in both cities.

Patients whose sputum samples were sent to the study laboratories for diagnosis of pulmonary TB between 4 February and 4 October 2012 were eligible. There were no exclusion criteria, but in the Xpert arm, samples considered insufficient or inadequate for Xpert processing according to the manufacturer's guidance [23] were tested only by smear examination. A sputum sample was considered insufficient for Xpert testing if its volume was less than 1 ml, and was considered inadequate for Xpert testing if on macroscopic examination it did not contain sputum or was blood-stained (as this may inhibit the PCR reaction) [24].

### Study Design

**Figure 1:**
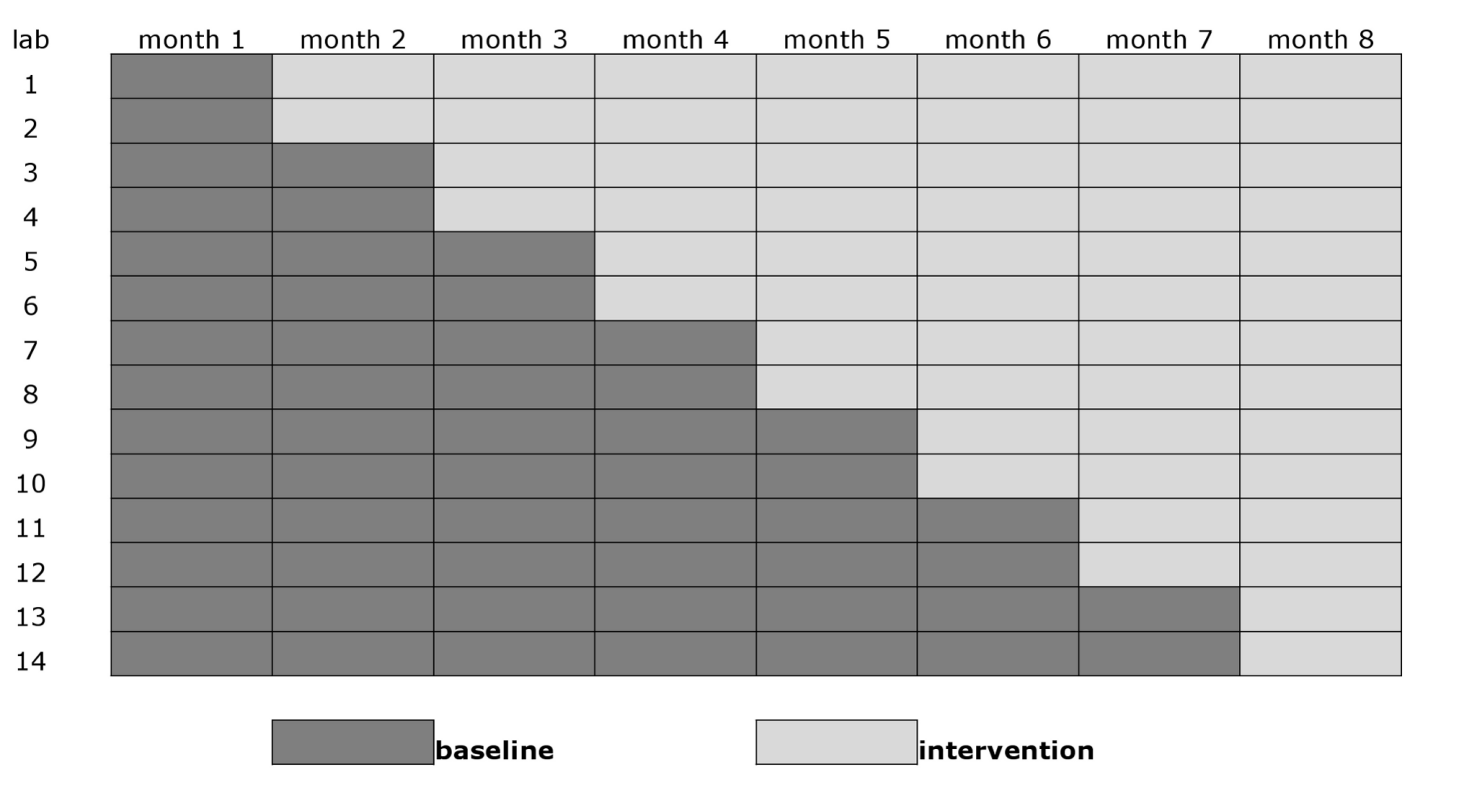
Stepped-wedge design with 14 clusters (study laboratories with serviced clinics) and eight monthly measurement periods.

This trial was a group-based comparison with phased introduction of Xpert to replace sputum smears as the initial diagnostic test for new pulmonary TB (Figure 1) [25]. A stepped-wedge design was chosen as it allowed a randomized comparison within a pilot project before national rollout. The units of comparison were TB laboratories and the clinics that use their services. The 14 trial laboratories were randomly assigned to the order in which they entered the intervention. To prevent imbalanced randomization with respect to important confounding variables as a result of the relatively small number of units, we applied restrained randomization based on the size of the monthly case load (low [*n* = 2], intermediate [*n* = 10], and high [*n* = 2]) of the laboratories, and on the estimated HIV prevalence (low [*n* = 12] and high [*n* = 2]) among the patients [26]. Allocation was not concealed, but laboratory staff and physicians were blinded to the order of entry into the intervention until Xpert was introduced.

In the smear microscopy arm, up to two sputum smears per patient were examined by conventional light microscopy based on direct Ziehl-Neelsen staining, as per routine. In the Xpert arm, usually only one of the submitted samples was examined. The second sample was processed for Xpert testing only if the first one was inadequate or insufficient, or if an error in processing occurred. If the first sample was processed successfully, the second sample was discarded. Results were reported back to the requesting clinic. Patients with an Xpert rifampicin-resistant result were referred to a referral center, and provisionally started on first-line treatment while awaiting confirmation by phenotypic DST, in line with existing Brazilian National TB Program guidelines (Löwenstein-Jensen medium or Mycobacteria Growth Indicator Tube [BD Microbiology Systems, Cockeysville, Maryland, US], according to the referral laboratory routine). This policy at the time of the study was based on the low drug resistance prevalence in Brazil and the expected low positive predictive value (PPV) of an Xpert rifampicin resistance result. Sample size was calculated based on a laboratory-confirmed TB notification rate of 50/100,000/year, an average cluster population of 500,000, a coefficient of variation of 0.25, and an additional design effect due to the cluster design of 1.5 [26],[27]. The study was powered to be able to detect a 60% increase in laboratory-confirmed pulmonary TB with a 5% type I error and an 80% type II error in the 8-mo study period.

All laboratories started off providing samples in the smear microscopy arm. Two laboratories then switched overnight to the Xpert arm every month, so that in the eighth (final) month of the trial, all units were in the Xpert arm (Figure 1). Fourth generation Xpert cartridges (G4) were used.

Primary endpoints were (1) the notification rate of laboratory-confirmed pulmonary TB to SINAN by any of the clinics relying on study laboratories' services, measured by the difference and the ratio of rates in the intervention versus the baseline period, and (2) time to treatment initiation, estimated by the notification date minus the laboratory result date. In Brazil, notification of TB to SINAN is mandatory, and is done at the time of treatment initiation, such that notification can be considered to indicate that, and when, a patient started treatment.

Secondary endpoints were the following notification rates: for pulmonary TB despite a negative test result, for pulmonary TB without any laboratory result reported, and for overall pulmonary TB irrespective of laboratory test result. Additional endpoints were the rate of Xpert tests positive for rifampicin resistance and the proportion of patients with a rifampicin-resistant Xpert result confirmed by conventional DST (PPV).

### Data Collection, Management, and Analysis

Data were collected from the routine laboratory reporting system (Gerenciamento de Ambiente Laboratorial [GAL]) and SINAN.

GAL contains details and results of all diagnostic tests ordered in the public laboratory system, entered by the laboratories. SINAN contains demographic and clinical data on all patients starting TB treatment, entered by the treating physicians or nurses. Entries in GAL were checked periodically for discrepancies against the regular TB laboratory logbooks and Xpert machines' logs; errors were corrected directly in GAL. When the sample collection period was completed, GAL records related to diagnostic testing were extracted and allocated to the smear microscopy or Xpert arm according to sample processing dates. For the smear microscopy arm, any (first or second) positive test was considered a positive result. Pulmonary TB notifications for the study period were extracted from SINAN and checked manually for inconsistencies.

For Rio de Janeiro, pulmonary TB cases notified by clinics outside the municipal primary care network were excluded. For Manaus, all notified pulmonary TB cases were included. Pulmonary TB cases in Manaus that were notified in SINAN but not identified in GAL were assigned to one of the three participating laboratories by adding to each laboratory a number of notified cases proportional to the number notified with laboratory confirmation, stratified by month, sex, and age group.

The databases were linked using RECLINK [28] by name, date of birth, and sex; additional manual linkage was performed using the following algorithm in Stata version 12 (Stata Corp, College Station, Texas, US): patients were considered identical if (1) sex, clinic, and date of birth were the same, and name was similar except for missing given names, abbreviations, or different spelling, or (2) sex and clinic were the same; name was similar except for missing given names, abbreviations, or different spelling; there was a 0- to 14-d difference in result report date in GAL and start of treatment according to SINAN; and date of birth differed for day only, month only, or year only, or the date and month were swapped (e.g., 11 April and 4 November).

Culture and DST results for patients with Xpert rifampicin-resistant samples were obtained from the Brazilian MDR TB reporting system by manual linkage [29].

Analyses were performed in Stata version 12. Numbers of laboratory diagnoses of TB and TB notifications were calculated for the smear microscopy and Xpert arms, stratified by municipality, age group, sex, HIV co-infection, and study month. Since the trial did not follow cohorts of patients, the units of analysis were not individual patients but populations with their number of notified cases. We therefore constructed an aggregated database of the number of TB notifications and population denominators for each of the 896 strata combining laboratory (*n* = 14), study month (*n* = 8), sex (*n* = 2), and age group (*n* = 4). For calculation of diagnostic and notification rates, population denominators took into account projected growth during the study period based on age- and sex-specific projected growth rates (separately for Rio de Janeiro and Manaus) [30] and were adjusted for variations in monthly number of days clinics were open by weighting the number of person-months for the proportion of patients with suspected TB with samples examined each month out of the total number examined by the laboratory during the whole study period, stratified by sex and age group.

The primary analyses were cluster-averaged, i.e., they compared the means of the cluster-specific notification rates between the 14 Xpert and the 14 smear microscopy cluster periods by their ratios and differences. Since the cluster-averaged method does not allow likelihood-based approaches for multivariable analysis, we adjusted the resulting rate ratio for potential confounding variables using a population-averaged quasi-likelihood method [26]. This method consisted of fitting a multivariable Poisson regression model that included all covariates except the notification rate (i.e., the endpoint of interest), and then comparing the model residuals for both trial arms by *t*-test or Wilcoxon rank sum test as appropriate [26]. The covariates included municipality (Rio de Janeiro or Manaus), age, sex, and rate of positive smear examinations observed in the first month, when all study laboratories were using smear examination. The last covariate was included to adjust for the baseline level of the endpoint parameter because with small numbers of randomization units (clusters), the randomization may not result in balanced distribution between the trial arms with respect to the expected endpoint. This cluster-averaged analysis approach provides the most robust results when the number of clusters is small, but has low statistical power [26]. Therefore, as a secondary analysis, we fitted mixed multilevel Poisson regression models to the overall aggregated data, specifying laboratory as the level of clustering to correct for within-laboratory correlation [31]. The multivariable models included municipality, age, sex, baseline smear positivity rate, and calendar time as covariates.

For all notified patients for whom a sputum sample had been submitted for laboratory testing, time to standard first-line treatment initiation was calculated as days between sputum processing date (obtained from GAL) and date of notification (obtained from SINAN) as a proxy for treatment initiation date. Cluster-averaged mean time intervals between sputum processing and start of treatment were compared with the Wilcoxon signed-rank test. For Xpert rifampicin-resistant cases, time to initiation of treatment with second-line drugs could be assessed only in the intervention arm.

We calculated the crude proportion of diagnostic samples that tested positive for rifampicin resistance by Xpert, as well as the PPV compared to DST. All notification rates are expressed per year.

We excluded any laboratory diagnosis made in the Xpert arm by smear examination, for two reasons. First, this approach would best reflect the situation of only Xpert being available, and therefore would best quantify the impact on TB notification of replacing smear examination with Xpert testing. Second, this approach would err on the conservative side with regard to the magnitude of increase in notification of laboratory-confirmed TB due to the use of Xpert. Unlike trials in which the endpoint is derived by dividing the number of diagnosed patients (numerator) by the number of tested patients (denominator), the endpoint in the present trial had as the denominator the population served by the study laboratories. Therefore, excluding patients diagnosed by smear microscopy in the intervention arm from the numerator did not affect the denominator, such that after excluding the smear-diagnosed patients, the notification rate for the intervention arm would by definition be lower than that without this exclusion, bringing the notification rate ratio for the intervention compared to the baseline arm closer to one. For the primary endpoints, we also show the intention to treat (ITT) analysis, including laboratory diagnoses made through smear examination in the Xpert arm.

## Results

**Figure 2:**
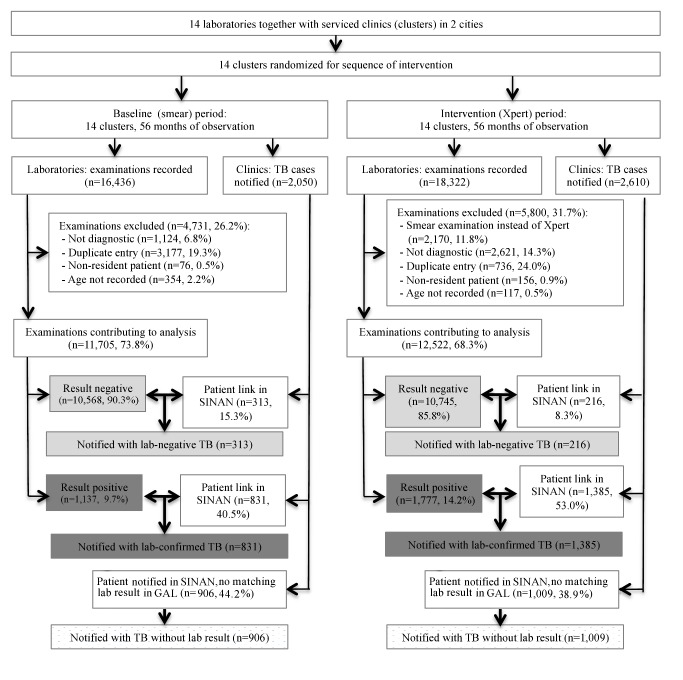
Flowchart showing study inclusion in baseline (smear examination) and intervention (Xpert MTB/RIF) arms. Bold arrows indicate cross-linkage between databases.

During the study period, the 14 laboratories examined 34,758 sputum specimens. Excluded were 4,731 (28.8%) specimens examined in the smear microscopy arm, and 5,800 (31.7%) examined in the Xpert arm, mostly those obtained for treatment follow-up and duplicate samples (Figure 2). The number of duplicate samples excluded was larger in the smear microscopy arm because often two samples per patient were examined. In total, 11,705 specimens in the smear microscopy arm and 12,522 in the Xpert arm were included in the primary analysis.

There were 1,137 (9.7%) positive smear examinations and 1,777 (14.2%) positive Xpert examinations (*p*<0.001; Table 1). The proportion of positive examinations varied among the laboratories from 3.4% to 15.4% for smears, and from 7.4% to 21.9% for Xpert, with a median increase of 60.5% (range 1.4% to 181.6%) in positivity when using Xpert.

**Table 1:** Numbers and characteristics of laboratory-reported and notified TB cases, by intervention arm.

Category	Baseline Arm (Smear Examination)						Intervention Arm (Xpert MTB/RIF)					
	Number of Tests Performed	Positive Test Result	Notified and Started on Treatment				Number of Tests Performed	Positive Test Result	Notified and Started on Treatment			
			Positive Test Result	Negative Test Result	No Test Result	All			Positive Test Result	Negative Test Result	No Test Result	All
**Total**	11,705	1,137 (9.7%)	831 (40.5%)	313 (15.3%)	906 (44.2%)	2,050 (100%)	12,522	1,777 (14.2%)	1,385 (53.1%)	216 (8.3%)	1,009 (38.7%)	2,610 (100%)
**Sex**												
Male	6,487	749 (65.9%)	555 (66.8)	202 (64.5%)	557 (61.5%)	1,314 (64.1%)	6,679	1,181 (66.5%)	910 (65.7%)	135 (62.5%)	637 (63.1%)	1,682 (64.4%)
Female	5,218	388 (34.1%)	276 (33.2)	111 (35.5%)	349 (38.5%)	736 (35.9%)	5,843	596 (33.5%)	475 (34.3%)	81 (37.5%)	372 (36.9%)	928 (35.6%)
**Age group**												
<15 y	400	34 (3.0%)	25 (3.0%)	3 (1.0%)	28 (3.1%)	56 (2.7%)	449	24 (1.4%)	14 (1.0%)	4 (1.9%)	38 (3.8%)	56 (2.1%)
15–39 y	4,786	603 (53.0%)	453 (54.5%)	165 (52.7%)	450 (49.7%)	1,068 (52.1%)	5,057	967 (54.4%)	768 (55.5%)	113 (52.3%)	514 (50.9%)	1,395 (53.4%)
40–59 y	4,228	371 (32.7%)	257 (30.9%)	108 (34.5%)	317 (35.0%)	682 (33.3%)	4,414	596 (33.5%)	455 (32.9%)	70 (32.4%)	310 (30.7%)	835 (32.0%)
≥60 y	2,291	129 (11.3%)	96 (11.6%)	37 (11.8%)	111 (12.2%)	244 (11.9%)	2,602	190 (10.7%)	148 (10.7%)	29 (13.4%)	147 (14.6%)	324 (12.4%)
**City**												
Rio de Janeiro	9,747	1,037 (91.2%)	756 (91.0%)	228 (72.8%)	755 (83.3%)	1,739 (84.8%)	6,989	1,171 (65.9%)	865 (62.5%)	81 (37.5%)	720 (71.8%)	1,666 (63.8%)
Manaus	1,958	100 (8.8%)	75 (9.0%)	85 (27.2%)	151 (16.7%)	311 (15.2%)	5,533	606 (34.1%)	520 (37.5%)	135 (62.5%)	289 (28.6%)	944 (36.2%)
**HIV status** a												
HIV positive	N/A	N/A	54 (6.5%)	58 (18.5%)	75 (9.9%)	187 (9.8%)	N/A	N/A	87 (6.3%)	50 (23.1%)	55 (5.5%)	192 (7.4%)
HIV negative	N/A	N/A	284 (34.2%)	97 (31.0%)	276 (36.5%)	657 (34.6%)	N/A	N/A	369 (26.6%)	51 (23.6%)	206 (20.4%)	626 (24.0%)
HIV unknown	N/A	N/A	493 (59.3%)	158 (50.5%)	405 (53.6%)	1,056 (55.6%)	N/A	N/A	929 (67.1%)	115 (53.2%)	748 (74.1%)	1,790 (68.6%)
**TB treatment history**												
New TB	N/A	N/A	688 (82.8%)	266 (85.0%)	0	954 (46.5%)	N/A	N/A	1,183 (85.4%)	194 (89.8%)	0	1,377 (52.8%)
Retreatment	N/A	N/A	142 (17.1%)	47 (15.0%)	0	189 (9.2%)	N/A	N/A	202 (14.6%)	15 (6.9%)	0	217 (8.3%)
Unknown	N/A	N/A	1 (0.1%)	0	906	907 (44.2%)	N/A	N/A	0	7 (3.2%)	1,009	1,016 (38.9%)

Percentages in parentheses are column percentages, except for total cases, for which the percentages are row percentages.

a Excluding 452 notified TB cases not linked to a specified study arm.

N/A, not available.

### Primary Endpoints

Over the study period, 4,660 patients were notified with pulmonary TB to SINAN by clinics served by the study laboratories. Of these, 2,216 patients (47.6%) could be linked to positive test results (76.0% of all 2,914 positive test results) and 529 (11.4%) to negative test results (2.5% of 21,234 negative results). The remaining 1,915 (41.1%) notified patients could not be linked to any study period test result (Figure 2). Conversely, 695 positive tests could not be linked to cases in the notification system, 303 (26.6% [95% CI = 24.0%, 29.2%]) in the baseline and 392 (22.1% [95% CI = 20.2%, 24.0%]) in the intervention arm (*p* = 0.003). We do not know whether these patients were treated without notification.

There was no difference in sex, age, or TB treatment history among these three groups (positive, negative, or no test results) or between the smear microscopy and Xpert arms. In both arms, HIV-infected patients were notified with laboratory-negative pulmonary TB more often than patients with negative or unknown HIV status were (Table 1).

Table 2 shows the unadjusted results for the notification rate of laboratory-confirmed TB (primary endpoint). The cluster-averaged laboratory-confirmed TB notification rate was 30.5/100,000/year in the smear microscopy arm versus 48.7/100,000/year in the Xpert arm, for a notification rate ratio of 1.59 (95% CI = 1.31, 1.88) and a notification rate difference of 18.2/100,000/year (95% CI = 9.4, 26.8) favoring the Xpert arm. Notification rates for laboratory-confirmed TB were higher for men than for women, and highest in the age group 15–59 y; these patterns were consistent across both arms. In the ITT analysis (Tables S1 and S2), which includes laboratory diagnosis by smear microscopy in the Xpert arm, the TB notification rate ratio was slightly higher: 1.67 (95% CI = 1.39, 1.96). The reasons for examining these 2,170 Xpert-arm specimens by smear microscopy were as follows: insufficient volume (1,151; 53.0%), inadequate material (e.g., saliva) (200; 9.2%), and logistical obstacles (819; 37.7%). The last referred to a single laboratory where the problems were solved after the first month of implementation.

**Table 2:** Notifications of laboratory-confirmed pulmonary TB by arm (baseline and intervention), by sex, age, municipality, and baseline smear-positive rate.

Category	Baseline Arm (Smear Examination)			Intervention Arm (Xpert MTB/RIF)			Notification Rate Ratio		Notification Rate Difference	
	Population (Person-Years)	Notification Rates (per 100,000 Population per Year)		Population (Person-Years)	Notification Rates (per 100,000 Population per Year)		Overall	Cluster-Averaged (95% CI)	Overall	Cluster-Averaged (95% CI)
		Overall^a^	Cluster-Averaged^b^ (95% CI)		Overall^a^	Cluster-Averaged^b^ (95% CI)				
**Total**	2,799,071	29.7	30.5 (24.9, 36.1)	2,647,008	52.3	48.7 (41.5, 55.8)	1.76	1.59 (1.31, 1.88)	22.6	18.1 (9.4, 26.8)
**Sex**										
**Male**	1,465,981	37.8	37.5 (30.7, 44.3)	1,404,926	64.7	60.1 (50.6, 69.5)	1.71	1.60 (1.31, 1.90)	26.9	22.6 (11.6, 33.7)
**Female**	1,333,090	20.7	23.1 (17.8, 28.5)	1,242,082	38.2	35.5 (28.0, 43.1)	1.85	1.54 (1.16, 1.92)	17.5	12.4 (3.6, 21.2)
**Age group**										
**<15 y**	594,291	4.2	3.7 (1.2, 6.2)	571,447	2.4	1.6 (0.6, 2.6)	0.58	0.43 (0.00, 1.11)	−1.8	−2.1 (−4.6, 0.4)
**15–39 y**	1,178,082	38.4	42.5 (31.8, 53.2)	1,079,807	71.2	65.0 (54.1, 75.7)	1.85	1.53 (1.19, 1.87)	32.7	22.5 (8.0, 36.9)
**40–59 y**	680,888	37.7	36.6 (26.8, 46.4)	640,892	71.1	67.8 (55.6, 79.9)	1.88	1.85 (1.45, 2.26)	33.3	31.1 (16.3, 46.0)
**≥60 y**	345,811	27.7	31.3 (19.2, 43.3)	354,862	41.7	43.7 (22.8, 64.5)	1.50	1.40 (0.66, 2.13)	14.0	12.4 (−10.5, 35.3)
**City**										
**Rio de Janeiro**	2,497,035	30.3	32.1 (25.8, 38.6)	1,725,565	50.1	48.5 (39.7, 57.3)	1.65	1.51 (1.19, 1.83)	19.8	16.4 (6.1, 16.3)
**Manaus**	302,036	24.8	24.6 (1.9, 47.1)	921,443	56.5	48.9 (19.2, 78.7)	2.27	1.99 (1.01, 2.98)	31.6	24.3 (0.3, 48.5)
**Baseline smear-positive rate** c										
**<27.5**	1,292,644	23.5	22.3 (15.6, 29.0)	1,241,463	50.4	41.1 (25.7, 56.6)	2.15	1.85 (1.22, 2.48)	26.9	18.9 (4.9, 33.0)
**27.5–36.4**	659,537	29.3	29.3 (20.8, 37.8)	471,307	51.8	54.4 (41.2, 67.6)	1.77	1.85 (1.44, 2.27)	22.5	25.1 (13.0, 37.2)
**≥36.5**	846,890	39.5	39.8 (30.2, 49.4)	934,238	55.1	51.5 (34.7, 68.1)	1.39	1.29 (0.89, 1.69)	15.6	11.7 (−4.4, 27.6)

a Overall notification rates: number of notified cases divided by population size, multiplied by 100,000.

b Cluster-averaged rates: mean of cluster-specific notification rates.

c Lab-specific rate of positive smear examinations in the first study month, per 100,000 population per year.

Thirteen of the 14 laboratories showed an increase in laboratory-confirmed TB notification rate with the switch to Xpert (Table 3), although the difference was not significant for five of these laboratories. The laboratory-specific notification rate ratios ranged significantly from 0.95 (95% CI = 0.65, 1.37) to 2.95 (95% CI = 1.48, 5.56), with a median of 1.53. Possible changes over time in the effectiveness of the intervention were examined by plotting the notification rate ratio of laboratory-confirmed TB against the number of months since the switch from smear examination to Xpert (Figure S2) and by plotting the difference of the notification rate ratio in the intervention and baseline arms (Figure S3). During any of the study months, the laboratory-confirmed TB notification rate based on Xpert exceeded that based on smear examination (Figure S3), and the notification rate for laboratory-confirmed TB based on Xpert remained stable over time (Figure S2). The notification rate for overall pulmonary TB increased for 11 laboratories and decreased for three, with the laboratory-specific notification rate ratio ranging from 0.76 to 1.75 (median 1.16; Figure S4).

**Table 3:** Notifications of pulmonary tuberculosis by laboratory according to arm (baseline versus intervention).

Laboratory	Baseline Arm (Smear Examination)			Intervention Arm (Xpert MTB/RIF)			Notification Rate Ratio (95% CI)	*p*-Value
	Population	Notifications	Notification Rate per 100,000 per Year (95% CI)	Population	Notifications	Notification Rate per 100,000 per Year (95% CI)		
1	71,136	27	38.0 (25.1, 55.2)	373,387	219	58.6 (51.1, 66.9)	1.55 (1.03, 2.40)	0.026
2	19,631	6	30.5 (11.2, 66.6)	168,531	78	46.2 (36.6, 57.8)	1.51 (0.67, 4.25)	0.331
3	128,993	28	21.7 (14.5, 31.4)	289,819	121	41.7 (34.7, 49.9)	1.92 (1.27, 3.01)	0.001
4	196,975	57	29.0 (21.9, 37.5)	646,545	401	62.1 (56.1, 68.4)	2.14 (1.62, 2.88)	0.000
5	172,172	92	53.4 (43.1, 65.6)	248,345	171	68.9 (58.9, 79.9)	1.29 (0.99, 1.68)	0.048
6	85,430	12	14.0 (7.3, 24.6)	106,367	41	38.6 (27.6, 52.3)	2.74 (1.41, 5.74)	0.001
7	162,646	55	33.8 (25.4, 44.0)	133,615	65	48.7 (37.6, 62.1)	1.44 (0.99, 2.10)	0.047
8	166,765	62	37.2 (28.5, 47.7)	123,472	59	47.8 (36.4, 61.7)	1.29 (0.88, 1.87)	0.169
9	212,759	73	34.3 (26.9, 43.2)	162,631	53	32.6 (24.5, 42.6)	0.95 (0.65, 1.37)	0.779
10	296,101	93	31.4 (25.3, 38.4)	147,148	87	59.1 (47.3, 72.9)	1.88 (1.39, 2.55)	0.000
11	514,874	124	24.1 (20.1, 28.7)	116,858	37	31.6 (22.3, 43.7)	1.31 (0.89, 1.91)	0.151
12	366,373	82	22.4 (17.8, 27.7)	81,875	26	31.8 (20.7, 46.5)	1.42 (0.88, 2.23)	0.129
13	181,159	39	21.5 (15.3, 29.4)	22,012	14	63.6 (34.8, 106.7)	2.95 (1.48, 5.56)	0.002
14	224,056	81	36.1 (28.7, 44.9)	26,402	13	49.3 (26.2, 84.2)	1.36 (0.70, 2.46)	0.305
Total/average	2,799071	831	30.5 (24.9, 36.1)	2,647,008	1,385	48.7 (41.5, 55.8)	1.59 (1.31, 1.88)	<0.001

The numbers of the laboratories reflect the sequence of introduction of the Xpert intervention (two labs at a time).

**Figure 3:**
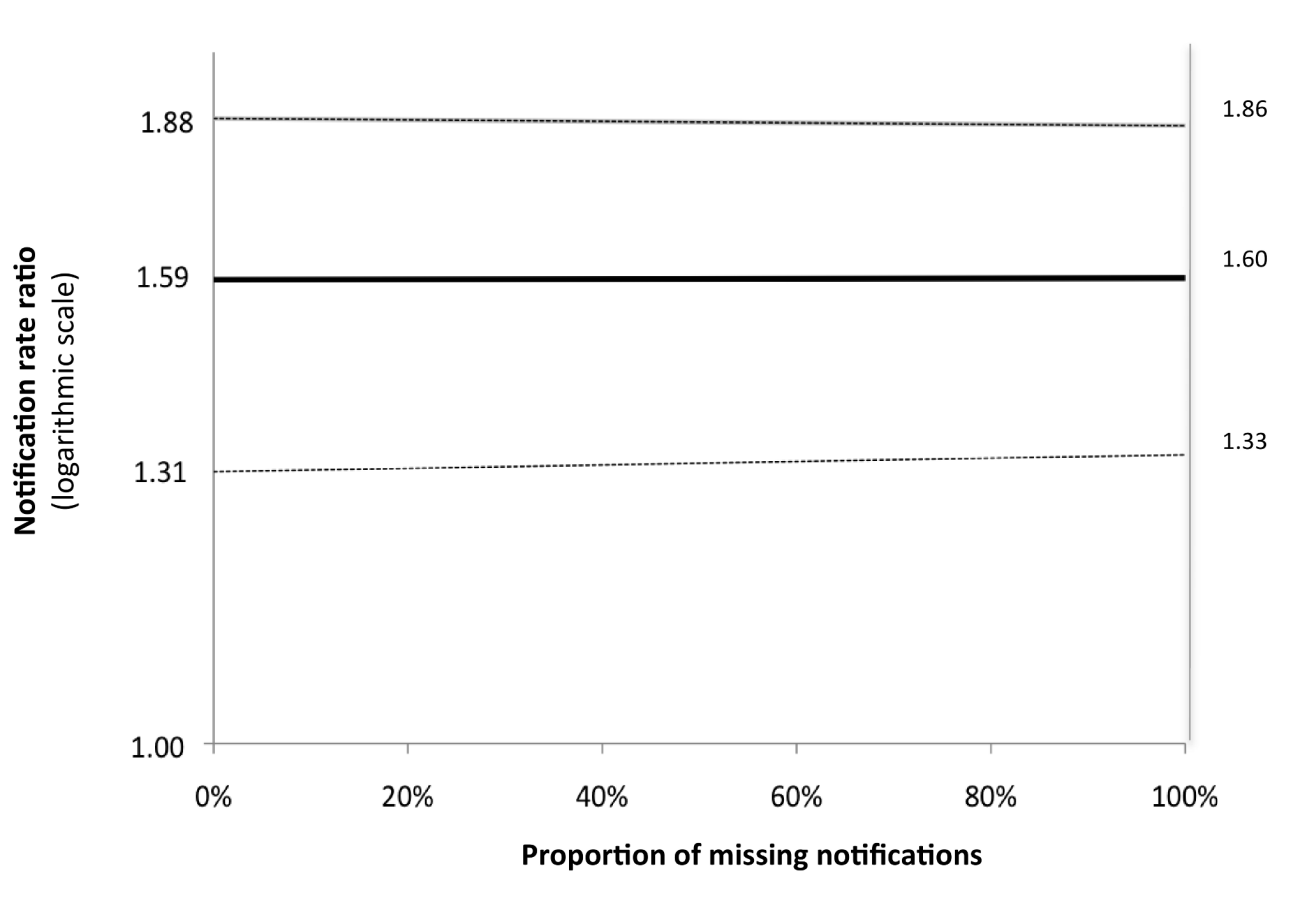
Sensitivity analysis for laboratory-confirmed TB diagnoses. Variation in unadjusted cluster-averaged notification rate ratio for laboratory-confirmed notifications, by proportion of missing notifications for laboratory-confirmed TB diagnoses that are due to failed linkage of records in the laboratory and notification databases.

We performed sensitivity analyses in which we assumed that an incrementing proportion of the laboratory-positive patients for whom no notification record was found was identified as “not notified” because of failed linkage between the GAL and SINAN databases. Of these analyses, the one assuming 100% failed database linkage equals the analysis comparing between the intervention and baseline arms the rates of positive laboratory tests irrespective of notification. There was no significant change in rate ratio (Figure 3). This means that the missing notifications of patients with positive laboratory results, whether because of failed database linkage or because of failed notification, occurred at random and did not bias our primary endpoints.

**Figure 4:**
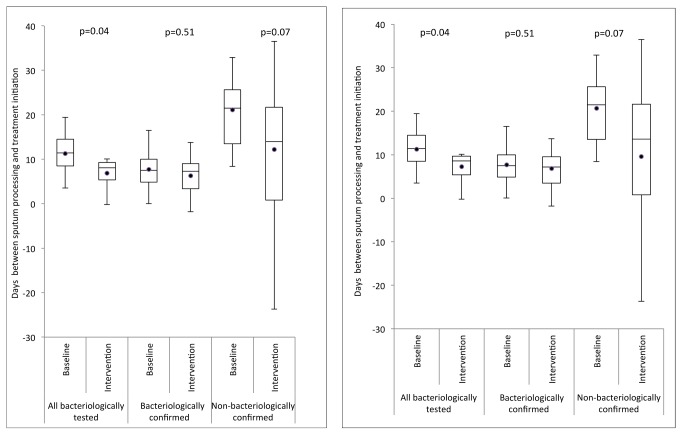
Box-and-whisker plot of cluster-averaged time interval between processing of sputum and start of first-line drug treatment in the baseline (smear examination) and intervention (Xpert MTB/RIF) arms. Delays are shown for three groups: (1) all TB patients notified for whom a sputum test was performed, (2) TB patients notified with bacteriological confirmation, and (3) TB patients notified without bacteriological confirmation. Left: per-protocol analysis; right: ITT analysis.

Treatment was initiated before sputum sample processing in 417 (36.5%) and 585 (36.5%) of those patients notified with a bacteriological test result in the smear microscopy and Xpert arms, respectively. Overall, the cluster-averaged time interval between sputum processing (generally the same day as sputum collection) and start of treatment decreased from a median of 11.4 d (interquartile range [IQR] = 8.5–14.5) in the smear microscopy arm to 8.1 d (IQR = 5.4–9.3) in the Xpert arm (*p* = 0.04; Figure 4, left) for the per-protocol analysis, and to 8.6 d (IQR 5.4–9.7, *p* = 0.04; Figure 4, right) for the ITT analysis. Stratification of time intervals by bacteriological confirmation status showed no decrease for laboratory-confirmed TB notifications (median 7.5 [IQR = 4.9–10.0] and 7.3 [IQR = 3.4–9.0], *p* = 0.51) or for laboratory-negative TB notifications (median 21.5 [IQR = 13.5–25.6] and 14.0 [IQR 0.8–21.7], *p* = 0.07).

### Secondary Endpoints

In contrast to the increase in the cluster-averaged laboratory-confirmed notification rate with the switch to Xpert, there were no significant changes in the cluster-averaged notification rate of laboratory-negative pulmonary TB (notification rate ratio 0.61, 95% CI = <0.01, 1.23), non-tested pulmonary TB (notification rate ratio 0.97, 95% CI = 0.63, 1.30), or overall pulmonary TB (notification rate ratio 1.15, 95% CI = 0.94, 1.37; Table 4). With multivariable adjustment, only the laboratory-negative TB notification rate ratio decreased significantly (0.52, 95% CI = 0.21, 0.84, *p* = 0.004; Table 4). Results for secondary endpoints stratified by patients' and laboratories' characteristics are presented in Tables 5–8. Across the laboratories, the population-based rates of positive test results regardless of notification (Figure S5) showed a distribution similar to that of the laboratory-confirmed TB notifications (Figure S4).

**Table 4:** Cluster-averaged notification rates, rate differences, and rate ratios for laboratory-confirmed TB, TB with negative test result, TB with no testing, and overall pulmonary TB.

Category	Notification Rate (95% CI)		Notification Rate Difference (95% CI)	Notification Rate Ratio (95% CI)			
	Baseline (Smear Microscopy)	Intervention (Xpert)		Unadjusted	*p*-Value	Adjusted^a^	*p*-Value
Laboratory-confirmed notifications	30.5 (24.9, 36.1)	48.7 (41.5, 55.8)	18.1 (9.4, 26.8)	1.59 (1.31, 1.88)	<0.001	1.59 (1.32, 1.87)	<0.001
Labboratory-confirmed notifications, ITT analysis^b^	30.5 (24.9, 36.1)	51.1 (44.0, 58.3)	20.6 (12.0, 29.2)	1.67 (1.39, 1.96)	<0.001	1.68 (1.41, 1.95)	<0.001
Notifications despite negative laboratory result	12.1 (6.1, 18.0)	7.3 (2.1, 12.5)	−4.8 (, 12.3, 2.8)	0.61 (<0.01, 1.23)	0.206	0.52 (0.21, 0.84)	0.004
Notifications with no laboratory test	36.9 (26.8, 47.1)	35.8 (27.6, 43.9)	−1.1 (−13.5, 11.2)	0.97 (0.63, 1.30)	0.850	0.98 (0.64, 1.32)	0.923
All notifications	79.6 (65.7, 93.4)	91.7 (80.2, 103.2)	12.2 (−5.0, 29.3)	1.15 (0.94, 1.37)	0.157	1.16 (0.96, 1.37)	0.115
Positive laboratory examinations	41.5 (34.1, 48.8)	66.2 (57.3, 75.2)	24.7 (13.7, 35.8)	1.60 (1.33, 1.86)	<0.001	1.62 (1.40, 1.84)	<0.001

Notification rate ratio is for intervention (Xpert) compared to baseline (smear examination) arm.

a Notification rate ratio adjusted for sex, age, municipality, and baseline smear-positive rate, quasi-likelihood population-averaged method.

b ITT analysis assuming availability of back-up smear examination.

**Table 5:** Notifications of pulmonary tuberculosis despite negative laboratory test.

Catagory	Baseline Period (Smear Examination)			Intervention Period (Xpert MTB/RIF)			Notification Rate Ratio	
	Population (Person-Years)	Notification Rate (per 100,000 Population per Year)		Population (Person-Years)	Notification Rate (per 100,000 Population per Year)		Overall	Cluster-Averaged (95% CI)
		Overall	Cluster-Averaged (95% CI)		Overall	Cluster-Averaged (95% CI)		
**Total**	2,799,071	11.2	12.1 (6.1, 18.0)	2,647,008	8.2	7.3 (2.1, 12.5)	0.73	0.61 (<0.01, 1.23)
**Sex**								
**Male**	1,465,981	13.7	15.1 (7.2, 22.9)	1,404,926	9.6	9.7 (2.0, 17.3)	0.70	0.64 (<0.01, 1.33)
**Female**	1,333,090	8.3	8.8 (3.8, 13.9)	1,242,082	6.5	4.9 (1.6, 8.1)	0.78	0.55 (<0.01, 1.20)
**Age group**								
**<15 y**	594,291	0.5	0.5 (0.0, 1.1)	571,447	0.7	0.0 (0.0, 0.6)	1.39	0.47 (<0.01, 1.75)
**15–39 y**	1,178,082	14.0	15.8 (7.5, 24.2)	1,079,807	10.5	9.2 (1.5, 16.9)	0.75	0.58 (<0.01, 1.26)
**40–59 y**	680,888	15.8	16.7 (6.5, 26.9)	640,892	10.9	11.4 (3.4, 19.3)	0.69	0.68 (<0.01, 1.42)
**≥60 y**	345,811	10.7	12.3 (3.2, 21.3)	354,862	8.2	9.3 (1.4, 17.2)	0.76	0.76 (<0.01, 1.69)
**City**								
**Rio de Janeiro**	2,497,035	9.1	9.3 (8.2, 10.3)	1,725,565	4.7	4.1 (2.4, 5.9)	0.51	0.45 (0.24, 0.66)
**Manaus**	302,036	28.1	22.5 (0.0, 76.5)	921,443	14.6	19.1 (0.0, 57.4)	0.52	0.85 (<0.01, 2.74)
**Baseline smear-positive rate** a								
**<27.5**	1,292,644	13.1	18.6 (0.0, 37.7)	1,241,463	11.2	11.9 (0.0, 38.7)	0.85	0.66 (<0.01, 1.82)
**27.5–36.4**	659,537	8.3	6.5 (0.0, 13.5)	471,307	7.0	4.7 (1.7, 7.8)	0.84	0.83 (<0.01, 1.97)
**≥36.5**	846,890	10.4	10.1 (7.5, 12.7)	934,238	4.7	6.7 (0.0, 13.6)	0.45	0.39 (0.10, 0.69)

Overall notification rates: number of notified cases divided by population size, multiplied by 100,000. Cluster-averaged rates: mean of cluster-specific notification rates.

a Laboratory-specific rate of positive smear examinations in the first study month, per 100,000 population per year.

**Table 6:** Notifications of pulmonary tuberculosis without recorded laboratory test.

Catagory	Baseline Period (Smear Examination)			Intervention Period (Xpert MTB/RIF)			Notification Rate Ratio	
	Population (Person-Years)	Notification Rate (per 100,000 Population per Year)		Population (Person-Years)	Notification Rate (per 100,000 Population per Year)		Overall	Cluster-Averaged (95% CI)
		Overall	Cluster-Averaged (95% CI)		Overall	Cluster-Averaged (95% CI)		
**Total**	2,799,071	32.4	36.9 (26.8, 47.1)	2,647,008	38.1	35.8 (27.6, 43.9)	1.18	0.61 (<0.01, 1.23)
**Sex**								
**Male**	1,465,981	38.1	45.5 (32.2, 58.8)	1,404,926	45.3	43.8 (34.7, 52.8)	1.19	0.64 (<0.01, 1.33)
**Female**	1,333,090	26.2	28.2 (19.0, 37.4)	1,242,082	30.0	26.8 (18.9, 34.7)	1.14	0.55 (<0.01, 1.20)
**Age group**								
**<15 y**	594,291	4.7	4.8 (1.1, 8.5)	571,447	6.6	5.8 (2.4, 9.2)	1.41	0.47 (<0.01, 1.75)
**15–39 y**	1,178,082	38.3	46.2 (29.9, 62.5)	1,079,807	47.7	46.6 (36.9, 56.3)	1.25	0.58 (<0.01, 1.26)
**40–59 y**	680,888	46.6	51.5 (34.7, 68.3)	640,892	48.4	43.8 (33.2, 54.4)	1.04	0.68 (<0.01, 1.42)
**≥60 y**	345,811	32.1	35.5 (18.4, 52.7)	354,862	41.4	39.9 (23.6, 56.2)	1.29	0.76 (<0.01, 1.69)
**City**								
**Rio de Janeiro**	2,497,035	30.2	34.3 (21.7, 46.8)	1,725,565	41.7	37.6 (27.3, 47.9)	1.38	0.45 (0.24, 0.66)
**Manaus**	302,036	50.0	46.7 (29.2, 64.2)	921,443	31.4	29.2 (19.1, 39.2)	0.63	0.85 (<0.01, 2.74)
**Baseline smear-positive rate** a								
**<27.5**	1,292,644	27.3	34.4 (14.7, 54.1)	1,241,463	34.7	33.6 (26.4, 40.9)	1.27	0.66 (<0.01, 1.82)
**27.5–36.4**	659,537	35.3	38.7 (4.0, 73.2)	471,307	43.3	37.2 (0.0, 80.1)	1.23	0.83 (<0.01, 1.97)
**≥36.5**	846,890	38.0	38.0 (13.7, 62.2)	934,238	39.9	36.7 (27.3, 46.1)	1.05	0.39 (0.10, 0.69)

Overall notification rates: number of notified cases divided by population size, multiplied by 100,000. Cluster-averaged rates: mean of cluster-specific notification rates.

a Laboratory-specific rate of positive smear examinations in the first study month, per 100,000 population per year.

**Table 7:** Notifications of overall pulmonary tuberculosis, regardless of test result.

Category	Baseline Period (Smear Examination)			Intervention Period (Xpert MTB/RIF)			Notification Rate Ratio		Notification Rate Difference	
	Population (Person-Years)	Notification Rate (per 100,000 Population per Year)		Population (Person-Years)	Notification Rate (per 100,000 Population per Year)		Overall	Cluster-Averaged (95% CI)	Overall	Cluster-Averaged (95% CI)
		Overall	Cluster-Averaged (95% CI)		Overall	Cluster-Averaged (95% CI)				
**Total**	2,799,071	73.2	79.6 (65.7, 93.4)	2,647,008	98.6	91.7 (80.2, 103.2)	1.35	1.15 (0.94, 1.37)	25.3	12.2 (−5.0, 29.3)
**Sex**										
**Male**	1,465,981	89.7	97.9 (81.3, 114.7)	1,404,926	119.7	113.5 (98.2, 128.7)	1.34	1.16 (0.94, 1.38)	30.1	15.6 (−6.0, 37.0)
**Female**	1,333,090	55.2	60.1 (47.3, 73.0)	1,242,082	74.7	67.2 (56.6, 77.7)	1.35	1.12 (0.85, 1.38)	19.5	7.1 (−8.8, 22.9)
**Age group**										
**<15 y**	594,291	9.4	9.0 (4.0, 14.0)	571,447	9.8	7.6 (3.8, 11.4)	1.04	0.84 (0.18, 1.51)	0.4	−1.4 (−7.4, 4.6)
**15–39 y**	1,178,082	90.6	104.5 (83.3, 125.8)	1,079,807	129.2	120.7 (104.4, 137.0)	1.43	1.15 (0.91, 1.40)	38.6	16.2 (−9.4, 41.5)
**40–59 y**	680,888	100.1	104.8 (77.6, 131.8)	640,892	130.3	122.9 (101.6, 144.3)	1.30	1.17 (0.86, 1.49)	30.2	18.1 (−14.6, 51.0)
**≥60 y**	345,811	70.6	79.0 (49.3, 108.6)	354,862	91.3	92.8 (55.4, 130.3)	1.29	1.18 (0.60, 1.75)	20.7	13.9 (−31.6, 59.3)
**City**										
**Rio de Janeiro**	2,497,035	69.6	75.7 (59.5, 92.0)	1,725,565	96.6	90.3 (75.9, 104.5)	1.39	1.19 (0.92, 1.46)	27.0	14.6 (−5.7, 34.8)
**Manaus**	302,036	102.9	93.8 (40.5, 147.0)	921,443	102.4	97.2 (58.4, 135.9)	1.00	1.04 (0.58, 1.49)	−0.5	3.4 (−39.1, 45.9)
**Baseline smear-positive rate** a										
**<27.5**	1,292,644	63.9	75.3 (39.9, 110.7)	1,241,463	96.4	87.0 (62.8, 111.3)	1.51	1.16 (0.68, 1.63)	32.5	11.7 (−24.0, 47.5)
**27.5–36.4**	659,537	72.9	74.5 (33.6, 115.5)	471,307	102.1	97.1 (57.1, 137.1)	1.40	1.30 (0.71, 1.90)	29.2	22.6 (−21.4, 66.7)
**≥36.5**	846,890	88.0	87.8 (61.9, 113.8)	934,238	99.9	92.1 (68.0, 116.2)	1.14	1.05 (0.71, 1.38)	11.9	4.3 (−25.2, 33.6)

Overall notification rates: number of notified cases divided by population size, multiplied by 100,000. Cluster-averaged rates: mean of cluster-specific notification rates.

a Laboratory-specific rate of positive smear examinations in the first study month, per 100,000 population per year.

**Table 8:** Positive laboratory test result for tuberculosis, irrespective of notification.

Category	Baseline Period (Smear Examination)			Intervention Period (Xpert MTB/RIF)			Notification Rate Ratio		Notification Rate Difference	
	Population (Person-Years)	Notification Rate (per 100,000 Population per Year)		Population (Person-years)	Notification Rate (per 100,000 Population per Year)		Overall	Cluster-Averaged (95% CI)	Overall	Cluster-Averaged (95% CI)
		Overall	Cluster-Averaged (95% CI)		Overall	Cluster-Averaged (95% CI)				
**Total**	2,799,071	44.3	46.0 (38.6, 54.8)	2,647,008	66.2	66.2 (56.5, 74.8)	1.49	1.41 (1.16, 1.66)	21.9	19.1 (7.4, 30.7)
**Sex**										
**Male**	1,465,981	55.8	57.3 (47.7, 66.9)	1,404,926	83.5	82.0 (70.1, 94.0)	1.50	1.43 (1.18, 1.69)	27.7	24.7 (10.2, 39.4)
**Female**	1,333,090	31.8	35.2 (28.2, 42.1)	1,242,082	46.7	46.8 (37.8, 55.7)	1.47	1.33 (0.63, 1.64)	14.9	11.7 (0.9, 22.4)
**Age group**										
**<15 y**	594,291	4.4	3.5 (1.2, 5.8)	571,447	6.3	5.9 (2.5, 9.3)	1.44	1.69 (0.57, 2.81)	1.9	2.4 (−1.5, 6.3)
**15–39 y**	1,178,082	55.8	60.6 (48.2, 73.0)	1,079,807	87.9	87.2 (73.4, 101.0)	1.57	1.44 (1.15, 1.73)	32.0	26.6 (9.0, 44.3)
**40–59 y**	680,888	63.7	65.5 (52.1, 79.0)	640,892	92.4	92.2 (78.8, 105.6)	1.45	1.41 (1.13, 1.68)	28.6	26.8 (8.7, 44.8)
**≥60 y**	345,811	35.9	40.9 (27.5, 54.4)	354,862	50.4	52.6 (30.9, 74.3)	1.41	1.29 (0.69, 1.88)	14.6	11.7 (−12.7, 36.0)
**City**										
**Rio de Janeiro**	2,497,035	45.1	48.8 (39.5, 58.0)	1,725,565	67.7	68.3 (27.3, 79.1)	1.50	1.40 (1.12, 1.67)	22.5	19.5 (6.1, 32.9)
**Manaus**	302,036	37.1	38.7 (3.9, 73.5)	921,443	63.5	56.1 (19.1, 88.8)	1.71	1.45 (0.65, 2.25)	26.5	17.4 (−13.4, 48.3)
**Baseline smear-positive rate** a										
**<27.5**	1,292,644	35.0	33.5 (25.2, 41.6)	1,241,463	58.9	51.2 (38.4, 63.9)	1.68	1.53 (1.16, 1.91)	23.9	17.8 (5.2, 30.4)
**27.5–36.4**	659,537	54.5	55.6 (46.6, 64.7)	471,307	69.2	67.8 (51.6, 84.0)	1.27	1.22 (0.94, 1.50)	14.6	12.2 (−3.3, 27.6)
**≥36.5**	846,890	50.8	51.8 (26.3, 77.4)	934,238	80.4	81.0 (68.6, 93.3)	1.58	1.56 (1.14, 1.98)	29.6	29.2 (7.4, 51.0)

Overall notification rates: number of notified cases divided by population size, multiplied by 100,000. Cluster-averaged rates: mean of cluster-specific notification rates.

a Laboratory-specific rate of positive smear examinations in the first study month, per 100,000 population per year.

### Secondary Analyses

Multivariable adjustment of the mixed multilevel models resulted in some increase in notification rate ratios for all endpoints (Table S3). The model for laboratory-confirmed TB showed a significant interaction between trial arm and baseline smear-positive rate: the notification rate ratio for Xpert versus smear microscopy decreased from 1.97 (95% CI = 1.58, 2.46) in laboratories with the lowest baseline smear-positive rates to 1.28 (95% CI = 1.01–1.61) in laboratories with the highest baseline rates (Table S4). The decreased notification rate ratio for laboratory-confirmed TB with increasing smear positivity rate was not associated with the proportion of smears graded as scanty (i.e., 1–9 bacilli per 100 fields). Restricting the analyses to the period during which laboratories contributed to both arms (months 2 to 7) also did not affect the results (Table S5; Figure S4).

### Additional Endpoints

Among 1,777 positive Xpert results, 66 (3.8%, 95% CI = 3.0, 4.7) were also positive for rifampicin resistance. Of these, 61 were notified in SINAN, including 45/1,377 (3.3%, 95% CI = 2.4, 4.3) new patients and 16/217 (7.4%, 95% CI = 4.3, 11.7) retreatment patients. For 50 of the 66 positive results for rifampin resistance (76%), a specimen was cultured in the reference laboratories, and DST results were available for 41 (66%). Rifampicin resistance was confirmed by phenotypic DST in 40/41 (PPV 98%; 95% CI = 87%, 99%), and MDR TB in 35/41 (PPV 85%, 95% CI = 71%, 94%). The time interval between processing Xpert and starting MDR TB treatment could be assessed for 27 patients with confirmed MDR TB and was a median 120 d (IQR = 88, 164).

## Discussion

This pragmatic trial showed that in a setting where laboratory diagnosis for pulmonary TB is largely restricted to sputum smear examination, implementing Xpert on a single sputum specimen increased laboratory-confirmed TB rates by 59% (31%–88%) and reduced time to treatment initiation from 11 to 8 d. The increase in TB confirmation was robust to potential confounding as well as to potential selection bias due to non-linkage of laboratory and notification databases, and was sustained over the study period. However, the overall notification rate of pulmonary TB—regardless of test results—did not increase significantly. In addition, because of the characteristics of the test, Xpert could accurately and promptly detect rifampicin resistance, which necessitates second-line drug treatment. Even in this setting with low drug resistance prevalence, the PPV for rifampicin resistance was high, although more than one-third of patients had no confirmatory DST result. Since culture with phenotypic DST is not routinely done in the country, these resistant cases would probably only be detected after treatment failure.

Our findings suggest that in this primary care setting, Xpert placed in laboratories is more useful for confirming pulmonary TB than for increasing TB detection. TB confirmation is relevant because it can potentially prevent many patients with respiratory symptoms who do not have TB from receiving unnecessary treatment and from having their true diagnosis delayed, although this remains to be demonstrated. The absence of a significant effect on detection of overall TB, a finding also recently reported from sub-Saharan Africa [4],[32], is probably largely explained by the large proportion of patients who were started on empirical treatment [33]. Empirical treatment for TB is a medical decision that depends in pretest probability, the patient's clinical condition, and test availability. The sensitivity and specificity of empirical treatment based on the WHO algorithm [34] varies substantially [35]. The pooled specificity for this algorithm in smear-negative patients was 69% in a meta-analysis [35], suggesting that a great number of patients falsely diagnosed with TB on clinical and radiological grounds could benefit from better diagnosis. In our study, 44.2% (95% CI = 43.9%, 47.9%) of patients in the smear microscopy arm and 38.7% (95% CI = 37.2%, 47.8%) in the Xpert arm were notified to SINAN as TB cases without a laboratory test, and an additional 15.3% (95% CI = 14.7%, 15.9%) and 8.3% (95% CI = 7.8%, 8.8%), respectively, with a negative test result. These groups of patients contain an unknown proportion of individuals who do not have TB, and this proportion ideally could be reduced by using Xpert. Due to Xpert's higher sensitivity, health-care workers can more confidently withhold TB treatment when the test result is negative, in particular for patients with no HIV infection, requesting the patient to come back for further TB diagnostics if the symptoms remain. We have no data to show how much extra delay to treatment initiation this would entail. The study period may have been too short to expect such a change in health-care workers' behavior, thus limiting our ability to show this possible benefit of Xpert.

There are alternative explanations for at least some of the non-laboratory-confirmed notifications. A positive test result may have been issued outside the study laboratories, such as at hospitals or small primary care laboratories, or database linkage may have failed despite the various algorithms used to address the lack of unique patient identifiers. Indeed, incomplete linkage has been described in previous studies in Brazil [36],[37]. Finally, dropout between diagnosis and treatment may be underreported [38]. Incomplete linkage and notification are also likely causes of the high proportion of positive laboratory results for which no disease notification could be found [36],[37]. We have no details about the patients who had a positive test but could not be retrieved in the notification database. The crude proportion of these missing patients decreased from the baseline to the intervention period. The rate ratio for laboratory-positive notifications (1.59, 95% CI = 1.31, 1.88; Figure 4) was equal to the rate ratio for positive laboratory results (1.60, 95% CI = 1.33, 1.86; Figure S5), and we observed similarity in age and sex distribution between patients with and without laboratory results, both suggesting that missing data happened at random. Together these data suggest that dropout between diagnosis and treatment was lower in the intervention than in the baseline arm, although the true magnitude of the difference cannot be established. Although initial loss to follow-up declined, it still was substantial, and likely added to transmission in the community. However, Xpert implementation has the potential to diminish transmission by reducing time to treatment initiation and initial loss to follow-up.

Despite its limitations, reliance on routine reporting data allowed a highly pragmatic trial design, closely resembling routine clinical practice. In particular, it obviated the need for individual informed consent, which would have made a trial of this size unfeasible and would have carried a risk of non-participation. In addition, the stepped-wedge trial design had the advantage of allowing an assessment of the effectiveness of this diagnostic intervention during its implementation with a limited number of laboratories, while a parallel cluster-randomized design would have required a larger number of randomization units [26]. The design has, however, potential for bias, in particular when assignment of the outcome to a study arm is not straightforward, such as with delayed treatment effects, or when conditions that affect the outcome change over time [39]. We believe that neither possibility for bias applies to our study. The primary endpoint of disease notification occurred within weeks after the diagnostic test, so that mis-assignment is unlikely.

Because of the small number of clusters in our study, we opted for the most robust and conservative statistical approach based on cluster-averaged rather than overall rates [26]. This primary analysis did not allow adjustment for time effects. An increase in effectiveness over time after the switch to Xpert could indicate a learning curve effect, while a decrease over time would suggest that the excess cases notified in the Xpert arm compared to the smear microscopy arm reflect detection of a temporary “backlog” of prevalent TB cases not identified by smear examination, rather than recent incident cases. However, Figures S1 and S2 show no consistent pattern of change with time since the start of using Xpert, making it unlikely that these effects occurred. Furthermore, the secondary analysis based on overall rates supported the primary analysis results, and adjustment for time effects only increased the effect of Xpert on notification rates.

Another possible source of bias was the unexpected high proportion of insufficient-volume samples in the Xpert arm, which had to be examined microscopically. However, the ITT analysis in which such smear examinations, when positive, were included only increased the effect of Xpert, and to limited extent. Indeed, recent data suggest that Xpert sensitivity is unaffected by sputum volume [40].

There was substantial variation in notification rate ratios for laboratory-confirmed TB across the 14 study laboratories. The largest relative and absolute increases were observed for the laboratories with relatively low notification rates in the smear microscopy arm. Since the sensitivity of Xpert is less operator-dependent than that of smear examination [3], especially for specimens with low bacterial load, these differences between the laboratories probably reflect differences in the operator-dependent sensitivity of smear examination. This difference would imply that Xpert improves TB case detection most where smear laboratory performance is suboptimal, e.g., because of high workload or inexperienced technicians, even though this may not translate into improvement of case finding for reasons such as empirical treatment and dropout between diagnosis and treatment.

The relatively low proportion of Xpert rifampicin-resistant cases with culture and DST results available in the public sector calls for more active follow-up of those patients during scale-up. The high PPV of Xpert for rifampicin resistance in this low drug resistance setting confirms the very high specificity of the G4 test [41] and its high PPV in programmatic settings, as recently reported in South Africa [42]. The PPV may be even higher if we take into account that clinically and epidemiologically relevant rifampicin resistance may not be detected by phenotypic DST [43]. Moreover, the high PPV for rifampicin resistance indicates that appropriate treatment decisions could be made as soon as Xpert rifampicin resistance results are available. However, because of the small number of rifampicin-resistant cases in this study, the wide confidence intervals, and the large amount of missing information, caution in interpreting this finding is needed. Further investigation during the scale-up of Xpert in the country is recommended in order to determine the appropriateness of changing the regimen upon two consecutive Xpert tests positive for rifampicin resistance, as recently recommended by WHO [10]. Negative predictive values could not be estimated from our data because of the study design. Results for negative predictive values are conflicting: while a recent study from India [44] suggested that in that setting, Xpert may miss a substantial proportion of rifampicin-resistant cases, in South Korea, the negative predictive value of Xpert was high (94.9%) [45].

Both smear microscopy and Xpert in principle are same-day tests. In a recent pragmatic trial, more patients had a same-day diagnosis and treatment initiation with Xpert than with smear microscopy [4]. This can be relevant if same-day diagnosis prevents dropout from treatment initiation. In our study, despite the significant reduction in time to treatment of patients with drug-susceptible TB from 11 to 8 d, a delay of more than a week is still unacceptable for a same-day test. This delay could partly be explained by the need for transport of samples to the laboratory. Also, clinic routines in which patients are requested to come back after 1 or 2 wk to hear the results of diagnostic tests may not have been adjusted yet, shortly after introduction of Xpert. Comprehensive health-care interventions addressing all factors contributing to delays in treatment initiation, but also improvement of uptake of Xpert testing (i.e., reduction in treatment initiation without laboratory testing), are necessary if the population is to fully benefit from this new diagnostic technology, as found in South Africa [32]. We discuss operational implications in more detail in another article [46].

Studies having treatment outcomes as endpoints are needed to evaluate the possible effects of Xpert beyond its immediate advantages. The 59% (95% CI = 31%, 88%) increase in laboratory-confirmed diagnoses could have a substantial impact on patient adherence to TB treatment. From the patient's perspective, motivation may be higher to engage in a long-term treatment with documented evidence of the presence of *M. tuberculosis*.

In conclusion, this programmatic study showed the effectiveness of replacing smear microscopy with Xpert for TB case confirmation and reduction of time to treatment initiation at the population level. These results support the Brazilian Ministry of Health's decision to adopt Xpert as a replacement for smear microscopy in 92 municipalities that cover more than 55% of new TB cases countrywide. However, important challenges remain in order to take full advantage of the potential of this technology in pragmatic conditions, such as reducing more dramatically treatment initiation delays and avoiding unnecessary empirical treatment.

## Supporting Information

Figure S1 **Algorithms for TB investigation and treatment.** (A) Study algorithm for sputum sample processing in the intervention arm. (B) National algorithm for Xpert-based pulmonary TB investigation in Brazil. (C) National algorithm for smear-based pulmonary TB investigation in Brazil.
(TIF)

Figure S2 **Notification rates of laboratory-confirmed TB for the intervention arm, by month since start of using Xpert.** Dots denote notification rates based on Xpert. Solid line: linear trend for notification rates (decline 0.74/100,000/year for each month; correlation coefficient 0.262, *p* = 0.95). Vertical bars: 95% confidence intervals for the notification rates.
(TIF)

Figure S3 **Difference between intervention (Xpert) and baseline (smear examination) arm in cluster-averaged notification rates of laboratory-confirmed TB, by study month.** Point estimates represent cluster-averaged notification rate differences between intervention and baseline arms. Values greater than zero denote higher notification rates for intervention than for baseline. Vertical bars: 95% confidence intervals for the cluster-averaged notification rate differences. Horizontal bar: notification rate difference for entire study period (18.1/100,000/year). Month 1 and month 8 had baseline-only and intervention-only observations, respectively.
(TIF)

Figure S4 **Notification rates for baseline (smear examination) and intervention (Xpert) arms, by study laboratory.** Cluster-specific notification rates (i.e., of all clinics that use the services of a particular study laboratory) of overall TB irrespective of laboratory confirmation. Laboratory number corresponds to the sequence of transition from baseline (smear examination) to intervention (Xpert) arm. Laboratories 2, 4, and 6 were situated in Manaus, all others in Rio de Janeiro.
(TIF)

Figure S5 **Positivity rate per laboratory, irrespective of notification.** Rate per 100,000 population per year for positive laboratory diagnoses, irrespective of notification, per laboratory. Laboratory number corresponds to the sequence of transition from baseline (smear examination) to intervention (Xpert) arm. Laboratories 2, 4, and 6 were situated in Manaus, all others in Rio de Janeiro.
(TIF)

Table S1 **Numbers and characteristics of laboratory-reported and notified TB cases, by intervention arm, including 54 smear results in the intervention arm (ITT analysis).**
(DOCX)

Table S2 **Notifications of laboratory-confirmed pulmonary TB by arm (baseline and intervention), by sex, age, municipality, and baseline smear-positive rate, including 54 smear results in the intervention arm (ITT analysis).**
(DOCX)

Table S3 **Secondary analysis: unadjusted and multivariably adjusted notification rate ratios for laboratory-confirmed TB, TB with negative test result, TB with no testing, and overall pulmonary TB, using a mixed multilevel model.**
(DOCX)

Table S4 **Notification rate ratios of laboratory-confirmed TB adjusted for calendar time, comparing the Xpert to the smear microscopy arm, stratified by baseline smear-positive rate (time-adjusted mixed multilevel model).** The time-adjusted mixed multilevel model for laboratory-confirmed notifications showed significant interactions between intervention status and municipality, and between intervention status and baseline smear positivity rate. The interaction with municipality no longer contributed significantly to the model likelihood when the interaction with baseline rate was included in the model, whereas the interaction with baseline rate continued to contribute significantly (*p*<0.001) even when the interaction with municipality was included. Hence, we concluded that the underlying interaction was between intervention status and baseline smear positivity rate. This table shows that the notification rate ratios for laboratory-confirmed TB decreased from 1.97 in the lowest baseline category to 1.28 in the highest baseline category.
(DOCX)

Table S5 **Cluster-averaged analysis excluding month 1 and month 8.** Excluding the data for months 1 and 8, which related to baseline-only and intervention-only observations, respectively, did not affect the cluster-averaged notification rate ratio for laboratory-confirmed TB (1.60, 95% CI 1.25, 1.96, *p*<0.01), although the notification rate ratio adjusted by quasi-likelihood population-averaged analysis was lower (1.48, 95% CI 1.17, 1.79, *p*<0.01). These exclusions slightly increased the unadjusted and adjusted cluster-averaged notification rate ratios for overall TB.
(DOCX)

Text S1 **Trial protocol.**
(DOC)

Text S2 **CONSORT checklist.**
(DOC)

## Abbreviations

DST: drug susceptibility testing
GAL: Gerenciamento de Ambiente Laboratorial (Brazilian laboratory reporting system)
IQR: interquartile range
ITT: intention to treat
MDR: multidrug-resistant
PPV: positive predictive value
SINAN: Sistema de Informação de Agravos de Notificação (Brazilian national notification system)
TB: tuberculosis
WHO: World Health Organization
Xpert: Xpert MTB/RIF assay

## References

[1] Raviglione M, Marais B, Floyd K, Lönnroth K, Getahun H, et al (2012) Scaling up interventions to achieve global tuberculosis control: progress and new developments. Lancet 379: 1902–1913 10.1016/S0140-6736(12)60727-2 22608339

[2] Boehme CC, Nabeta P, Hillemann D, Nicol MP, Shenai S, et al (2010) Rapid molecular detection of tuberculosis and rifampin resistance. N Engl J Med 363: 1005–1015 10.1056/NEJMoa0907847 20825313 PMC2947799

[3] Boehme CC, Nicol MP, Nabeta P, Michael JS, Gotuzzo E, et al (2011) Feasibility, diagnostic accuracy, and effectiveness of decentralised use of the Xpert MTB/RIF test for diagnosis of tuberculosis and multidrug resistance: a multicentre implementation study. Lancet 377: 1495–1505 10.1016/S0140-6736(11)60438-8 21507477 PMC3085933

[4] Theron G, Zijenah L, Chanda D, Clowes P, Rachow A, et al (2014) Feasibility, accuracy, and clinical effect of point-of-care Xpert MTB/RIF testing for tuberculosis in primary-care settings in Africa: a multicentre, randomised, controlled trial. Lancet 383: 424–435 10.1016/S0140-6736(13)62073-5 24176144

[5] Steingart KR, Schiller I, Horne DJ, Pai M, Boehme CC, et al (2014) Xpert® MTB/RIF assay for pulmonary tuberculosis and rifampicin resistance in adults. Cochrane Database Syst Rev 1: CD009593 10.1002/14651858.CD009593.pub3 PMC4470349 24448973

[6] Choi HW, Miele K, Dowdy D, Shah M (2013) Cost-effectiveness of Xpert® MTB/RIF for diagnosing pulmonary tuberculosis in the United States. Int J Tuberc Lung Dis 17: 1328–1335 10.5588/ijtld.13.0095 24025386 PMC3891798

[7] Menzies NA, Cohen T, Lin H-H, Murray M, Salomon JA (2012) Population health impact and cost-effectiveness of tuberculosis diagnosis with Xpert MTB/RIF: a dynamic simulation and economic evaluation. PLoS Med 9: e1001347 10.1371/journal.pmed.1001347 23185139 PMC3502465

[8] Vassall A, van Kampen S, Sohn H, Michael JS, John KR, et al (2011) Rapid diagnosis of tuberculosis with the Xpert MTB/RIF assay in high burden countries: a cost-effectiveness analysis. PLoS Med 8: e1001120 10.1371/journal.pmed.1001120 22087078 PMC3210757

[9] World Health Organization (2012) Tuberculosis diagnostics Xpert MTB/RIF test. WHO endorsement and recommendations. Available: http://www.who.int/tb/features_archive/factsheet_xpert_may2011update.pdf. Accessed 9 November 2013.

[10] World Health Organization (2011) Automated real-time nucleic acid amplification technology for rapid and simultaneous detection of tuberculosis and rifampicin resistance: Xpert MTB/RIF assay for the diagnosis of pulmonary and extrapulmonary TB in adults and children. Available: http://www.stoptb.org/wg/gli/assets/documents/WHO%20Policy%20Statement%20on%20Xpert%20MTB-RIF%202013%20pre%20publication%2022102013.pdf. Accessed 13 November 2014.

[11] Small PM, Pai M (2010) Tuberculosis diagnosis—time for a game change. N Engl J Med 363: 1070–1071. 20825320 10.1056/NEJMe1008496

[12] Schunemann HJ, Oxman AD, Brozek J, Glasziou P, Jaeschke R, et al (2008) Grading quality of evidence and strength of recommendations for diagnostic tests and strategies. BMJ 336: 1106–1110 10.1136/bmj.39500.677199.AE 18483053 PMC2386626

[13] Cobelens F, van den Hof S, Pai M, Squire SB, Ramsay A, et al (2012) Which new diagnostics for tuberculosis, and when? J Infect Dis 205 Suppl 2: S191–S198 10.1093/infdis/jis188 22476716

[14] Hanrahan CF, Selibas K, Deery CB, Dansey H, Clouse K, et al (2013) Time to treatment and patient outcomes among TB suspects screened by a single point-of-care Xpert MTB/RIF at a primary care clinic in Johannesburg, South Africa. PLoS ONE 8: e65421 10.1371/journal.pone.0065421 23762367 PMC3675091

[15] Yoon C, Cattamanchi A, Davis JL, Worodria W, den Boon S, et al (2012) Impact of Xpert MTB/RIF testing on tuberculosis management and outcomes in hospitalized patients in Uganda. PLoS ONE 7: e48599 10.1371/journal.pone.0048599 23139799 PMC3490868

[16] World Health Organization (2014) TB diagnostics and laboratory strengthening: WHO monitoring of Xpert MTB/RIF roll-out. Available: http://who.int/tb/laboratory/mtbrifrollout/en/. Accessed 31 October 2014.

[17] Zwarenstein M, Treweek S, Gagnier JJ, Altman DG, Tunis S, et al (2008) Improving the reporting of pragmatic trials: an extension of the CONSORT statement. BMJ 337: a2390. 19001484 10.1136/bmj.a2390 PMC3266844

[18] World Health Organization (2014) Global tuberculosis report 2014. Available: http://apps.who.int/iris/bitstream/10665/137094/1/9789241564809_eng.pdf?ua=1. Accessed 13 November 2014.

[19] Brasil Ministério da Saúde Secretaria de Vigilância Sanitária Programa Nacional de Controle da Tuberculose (2011) Manual de recomendações para o controle da tuberculose no Brasil. Available: http://www.cve.saude.sp.gov.br/htm/TB/mat_tec/manuais/MS11_Manual_Recom.pdf. Accessed 13 November 2014.

[20] Brasil Ministério da Saúde Secretaria de Vigilância em Saúde Programa Nacional de Controle da Tuberculose (2013) Programa Nacional de Controle da Tuberculose. Available: https://docs.google.com/file/d/0B0CE2wqdEaR-VG1fa0JJMi1qa0U/edit. Accessed 31 March 2014.

[21] Portal da Saúde (2013). Sistema de Informação de Agravos de Notificação—SINAN. O que é o SINAN. Available: http://dtr2004.saude.gov.br/sinanweb/. Accessed 4 July 2013.

[22] Portal da Saúde (2013) DATASUS: Informações de Saúde—demográficas e socioeconômicas [database]. Available: http://www2.datasus.gov.br/DATASUS/index.php?area=0206&VObj=http://tabnet.datasus.gov.br/cgi/deftohtm.exe?ibge/cnv/pop. Accessed 8 July 2013.

[23] Cepheid (n.d.) The new GeneXpert® system. Available: http://www.google.com.br/url?sa=t&rct=j&q=&esrc=s&source=web&cd=1&ved=0CB8QFjAA&url=http%3A%2F%2Fwww.cepheid.com%2Fus%2Fcomponent%2Fphocadownload%2Fcategory%2F1-about-us%3Fdownload%3D41%3Agx-brochure&ei=1S9lVLGKFYyagwSdkIQY&usg=AFQjCNHOvPFFlDOkUPcxH9BU2dXGI6ypxQ&sig2=BVKcXMgB2MT1g0G0m40zaQ&bvm=bv.79189006,d.eXY. Accessed 13 November 2014.

[24] Cepheid (2009) Xpert®MTB/RIF: two-hour detection of MTB and resistance to rifampicin. Available: http://tbevidence.org/documents/rescentre/sop/XpertMTB_Broch_R9_EU.pdf. Accessed 31 March 2014.

[25] Brown CA, Lilford RJ (2006) The stepped wedge trial design: a systematic review. BMC Med Res Methodol 6: 54 10.1186/1471-2288-6-54 17092344 PMC1636652

[26] Hayes RJ, Moulton LH (2009) Cluster randomized trials. Boca Raton (Florida): Chapman & Hall/CRC.

[27] Moulton LH, Golub JE, Durovni B, Cavalcante SC, Pacheco AG, et al (2007) Statistical design of THRio: a phased implementation clinic-randomized study of a tuberculosis preventive therapy intervention. Clin Trials 4: 190–199 10.1177/1740774507076937 17456522

[28] Camargo KR Jr, Coeli CM (2000) [Reclink: an application for database linkage implementing the probabilistic record linkage method.]. Cad Saude Publica 16: 439–447. 10883042 10.1590/s0102-311x2000000200014

[29] Sistema de Informação de Tratamentos Especiais de Tuberculose (2014) SITETB. Available: http://www.sitetb.org. Accessed 13 November 2014.

[30] Instituto Brasileiro de Geografia e Estatística (2013) Estimativas da população: metodologia adotada nas estimativas populacionais municipais. Available: http://www.ibge.gov.br/home/estatistica/populacao/estimativa2005/estimativa_pop.shtm. Accessed 5 December 2013.

[31] Hussey MA, Hughes JP (2007) Design and analysis of stepped wedge cluster randomized trials. Contemp Clin Trials 28: 182–191 10.1016/j.cct.2006.05.007 16829207

[32] Fielding KL, McCarthy K, Cox H, Erasmus L, Ginindza S, et al. (2014) Xpert as the first-line TB test in South Africa: yield, initial loss to follow-up, proportion treated [abstract]. 2014 Conference on Retroviruses and Opportunistic Infections; 3–6 March 2014; Boston, Massachusetts, US.

[33] Theron G, Peter J, Dowdy D, Langley I, Squire SB, et al (2014) Do high rates of empirical treatment undermine the potential effect of new diagnostic tests for tuberculosis in high-burden settings? Lancet Infect Dis 14: 527–532. 24438820 10.1016/S1473-3099(13)70360-8

[34] World Health Organization (2007) Improving the diagnosis and treatment of smear-negative pulmonary and extra-pulmonary tuberculosis among adults and adolescents: recommendations for HIV-prevalent and resource-constrained settings. Available: http://www.who.int/hiv/pub/tb/pulmonary/en/. Accessed 5 March 2014.

[35] Walusimbi S, Bwanga F, De Costa A, Haile M, Joloba M, et al (2013) Meta-analysis to compare the accuracy of GeneXpert, MODS and the WHO 2007 algorithm for diagnosis of smear-negative pulmonary tuberculosis. BMC Infect Dis 13: 507. 24172543 10.1186/1471-2334-13-507 PMC3833313

[36] Selig L, Guedes R, Kritski A, Spector N, Lapa E, Silva JR, et al (2009) Uses of tuberculosis mortality surveillance to identify programme errors and improve database reporting. Int J Tuberc Lung Dis 13: 982–988. 19723378

[37] Oliveira LM, PinheiroII RS (2011) Óbitos e internações por tuberculose não notificados no Município do Rio de Janeiro. Rev Saude Publica 45: 31–39.21181049

[38] Harries AD, Rusen ID, Chiang C-Y, Hinderaker SG, Enarson DA (2009) Registering initial defaulters and reporting on their treatment outcomes. Int J Tuberc Lung Dis 13: 801–803. 19555527

[39] Rhoda DA, Murray DM, Andridge RR, Pennell ML, Hade EM (2011) Studies with staggered starts: multiple baseline designs and group-randomized trials. Am J Public Health 101: 2164–2169 10.2105/AJPH.2011.300264 21940928 PMC3222403

[40] Theron G, Peter J, van Zyl-Smit R, Mishra H, Streicher E, et al (2011) Evaluation of the Xpert MTB/RIF assay for the diagnosis of pulmonary tuberculosis in a high HIV prevalence setting. Am J Respir Crit Care Med 184: 132–140 10.1164/rccm.201101-0056OC 21493734

[41] Foundation for Innovative New Diagnostics (2011) Performance of Xpert MTB/RIF version G4 assay. Geneva: Foundation for Innovative New Diagnostics. Available: http://www.stoptb.org/wg/gli/assets/documents/map/findg4cartridge.pdf. Accessed 2 February 2014.

[42] Osman M, Simpson JA, Caldwell J, Bosman M, Nicol MP (2014) GeneXpert MTB/RIF version G4 for identification of rifampin-resistant tuberculosis in a programmatic setting. J Clin Microbiol 52: 635–637 10.1128/JCM.02517-13 24478501 PMC3911341

[43] Van Deun A, Aung KJM, Bola V, Lebeke R, Hossain MA, et al (2013) Rifampin drug resistance tests for tuberculosis: challenging the gold standard. J Clin Microbiol 51: 2633–2640 10.1128/JCM.00553-13 23761144 PMC3719626

[44] Rufai SB, Kumar P, Singh A, Prajapati S, Balooni V, et al (2014) Comparison of Xpert MTB/RIF with line probe assay for detection of rifampin-monoresistant Mycobacterium tuberculosis. J Clin Microbiol 52: 1846–1852 10.1128/JCM.03005-13 24648554 PMC4042801

[45] Kwak N, Choi SM, Lee J, Park YS, Lee C-H, et al (2013) Diagnostic accuracy and turnaround time of the Xpert MTB/RIF assay in routine clinical practice. PLoS ONE 8: e77456 10.1371/journal.pone.0077456 24204834 PMC3812224

[46] Durovni B, Saraceni V, Cordeirodo-Santos M, Cavalcante SC, Soares E, et al (2014) Operational lessons drawn from pilot implementation of Xpert MTB/Rif in Brazil. Bull World Health Organ 92: 613–617. 25177076 10.2471/BLT.13.131409 PMC4147406

