# Correction: Impact of Replacing Smear Microscopy with Xpert MTB/RIF for Diagnosing Tuberculosis in Brazil: A Stepped-Wedge Cluster-Randomized Trial

**DOI:** 10.1371/journal.pmed.1001928

**Published:** 2015-12-03

**Authors:** Betina Durovni, Valeria Saraceni, Susan van den Hof, Anete Trajman, Marcelo Cordeiro-Santos, Solange Cavalcante, Alexandre Menezes, Frank Cobelens

During a secondary analysis, the authors discovered 147 duplicate entries in their study database that consists of over 30,000 entries. The duplication of entries was randomly distributed and after reanalysis only minor changes in effect measures, which do not impact the authors’ conclusions, were observed. For example, the notification rate ratio for laboratory confirmed TB (the study’s primary endpoint) was 1.59 (95% CI 1.31–1.88) in the original analysis and 1.60 (95% CI 1.31–1.89) in the revised analysis. A revised version of Table 4 is presented below.

**Table 4:** Cluster-averaged notification rates, differences and ratios for lab-confirmed TB, TB with negative test result, TB with no testing, and overall pulmonary TB.

	Notification rates (95% CI)		Notification rate difference (95% CI)	Notification rate ratios (95% CI)			
	Baseline (smear)	Intervention(Xpert)		unadjusted		adjusted^1^	
	NR	NR		NRR	*P*-value	NRR	*P*-value
Lab-confirmed notifications	29.9 (24.6–35.4)	47.9 (40.5–55.2)	18.0 (9.2–26.5)	1.60 (1.31–1.89)	<0.001	1.61 (1.29–1.93)	<0.001
Lab-confirmed notifications ITT^2^	29.9 (24.6–35.4)	50.4 (43.1–57.7)	20.4 (11.8–29.1)	1.68 (1.39–1.97)	<0.001	1.70 (1.38–2.02)	<0.001
Notifications despite negative lab result	12.1 (6.1–18.0)	7.3 (2.1–12.5)	-4.8 (-12.3–2.8)	0.61 (<0.01–1.23)	0.205	0.54 (0.21–0.83)	0.004
Notifications with no lab test	34.9 (25.3–44.5)	33.3 (28.3–39.8)	-1.6 (-13.3–10.0)	0.95 (0.62–1.29)	0.782	0.97 (0.64–1.32)	0.851
All notifications	77.0 (63.6–90.3)	88.5 (77.1–99.9)	11.4 (-5.1–28.2)	1.15 (0.93–1.37)	0.167	1.19 (0.97–1.35)	0.105
Positive laboratory examinations	40.9 (33.7–48.1)	65.5 (56.3–74.6)	24.6 (13.5–35.6)	1.60 (1.33–1.87)	<0.001	1.62 (1.40–1.84)	<0.001

NRR = notification rate ratio for intervention (Xpert MTB/RIF) compared to baseline (smear examination) arm. 95% CI = 95% confidence interval. TB = tuberculosis.

^1^ NRR adjusted for sex, age, municipality and baseline smear-positive rate, quasi-likelihood population-averaged method

^2^intention to treat (ITT) analysis assuming availability of back-up smear examination
