# Supplementary figures and images for: Impact of Replacing Smear Microscopy with Xpert MTB/RIF for Diagnosing Tuberculosis in Brazil: A Stepped-Wedge Cluster-Randomized Trial
Source: PLoS Med. 2014 Dec 9;11(12):e1001766. doi: 10.1371/journal.pmed.1001766 (PMC4260794; doi:10.1371/journal.pmed.1001766)

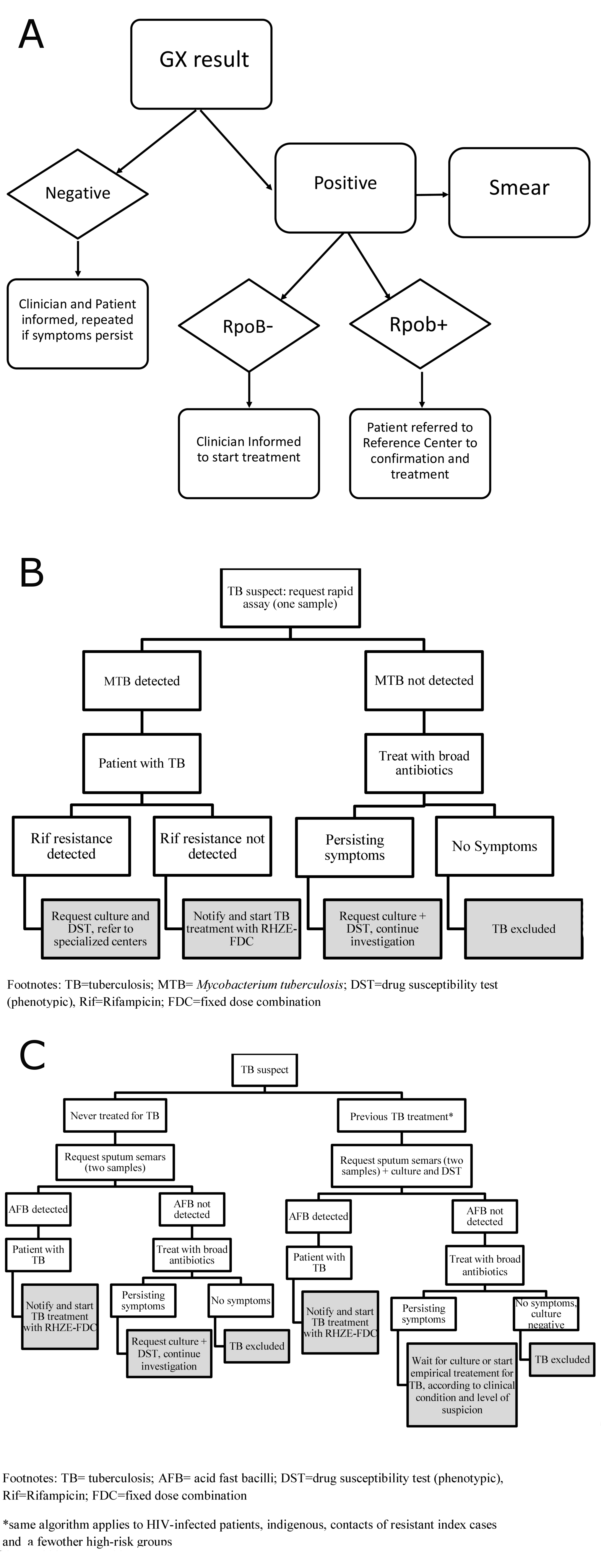

Supplement: Figure S1 — Algorithms for TB investigation and treatment. (A) Study algorithm for sputum sample processing in the intervention arm. (B) National algorithm for Xpert-based pulmonary TB investigation in Brazil. (C) National algorithm for smear-based pulmonary TB investigation in Brazil. (TIF) [file pmed.1001766.s001.tif]

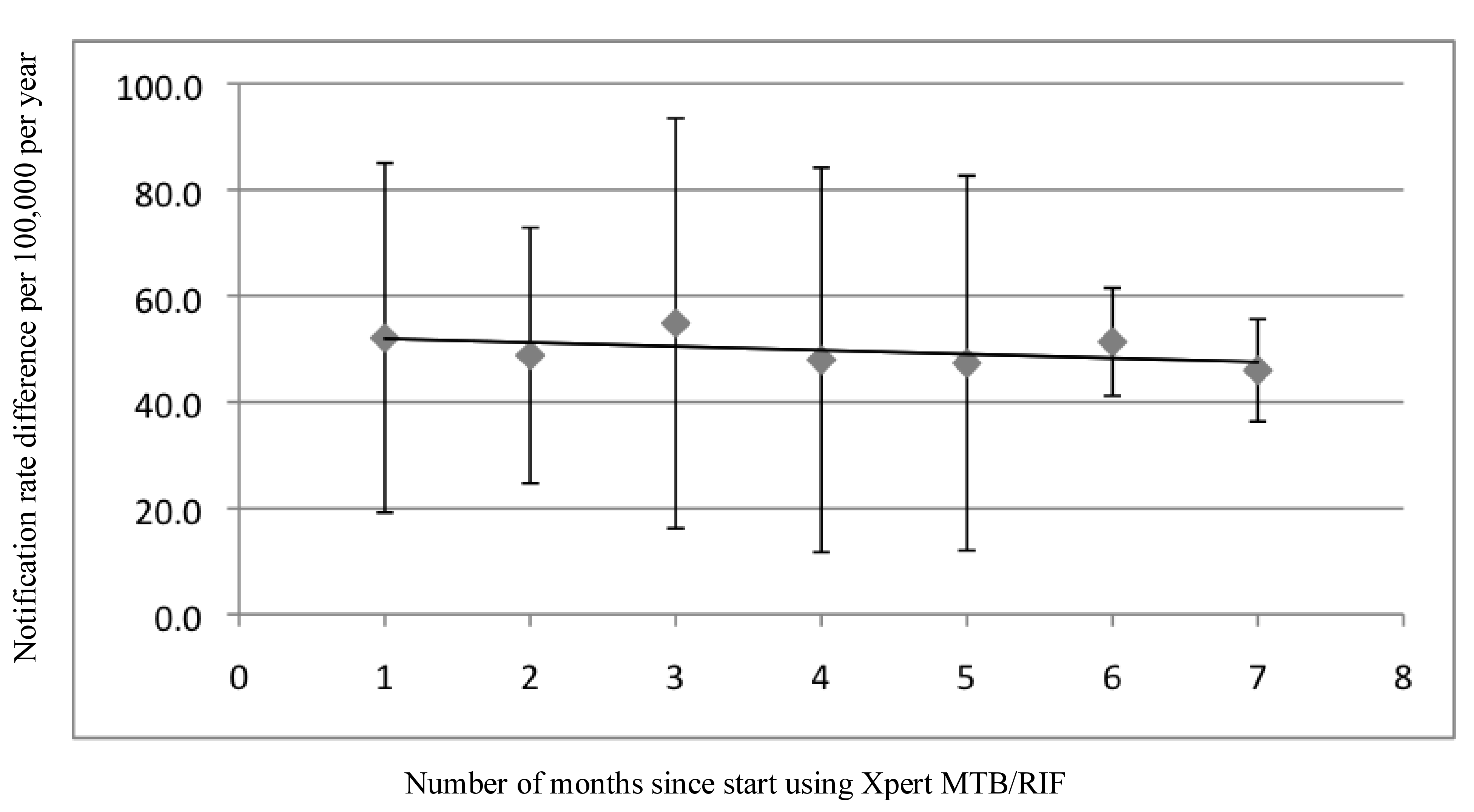

Supplement: Figure S2 — Notification rates of laboratory-confirmed TB for the intervention arm, by month since start of using Xpert. Dots denote notification rates based on Xpert. Solid line: linear trend for notification rates (decline 0.74/100,000/year for each month; correlation coefficient 0.262, p = 0.95). Vertical bars: 95% confidence intervals for the notification rates. (TIF) [file pmed.1001766.s002.tif]

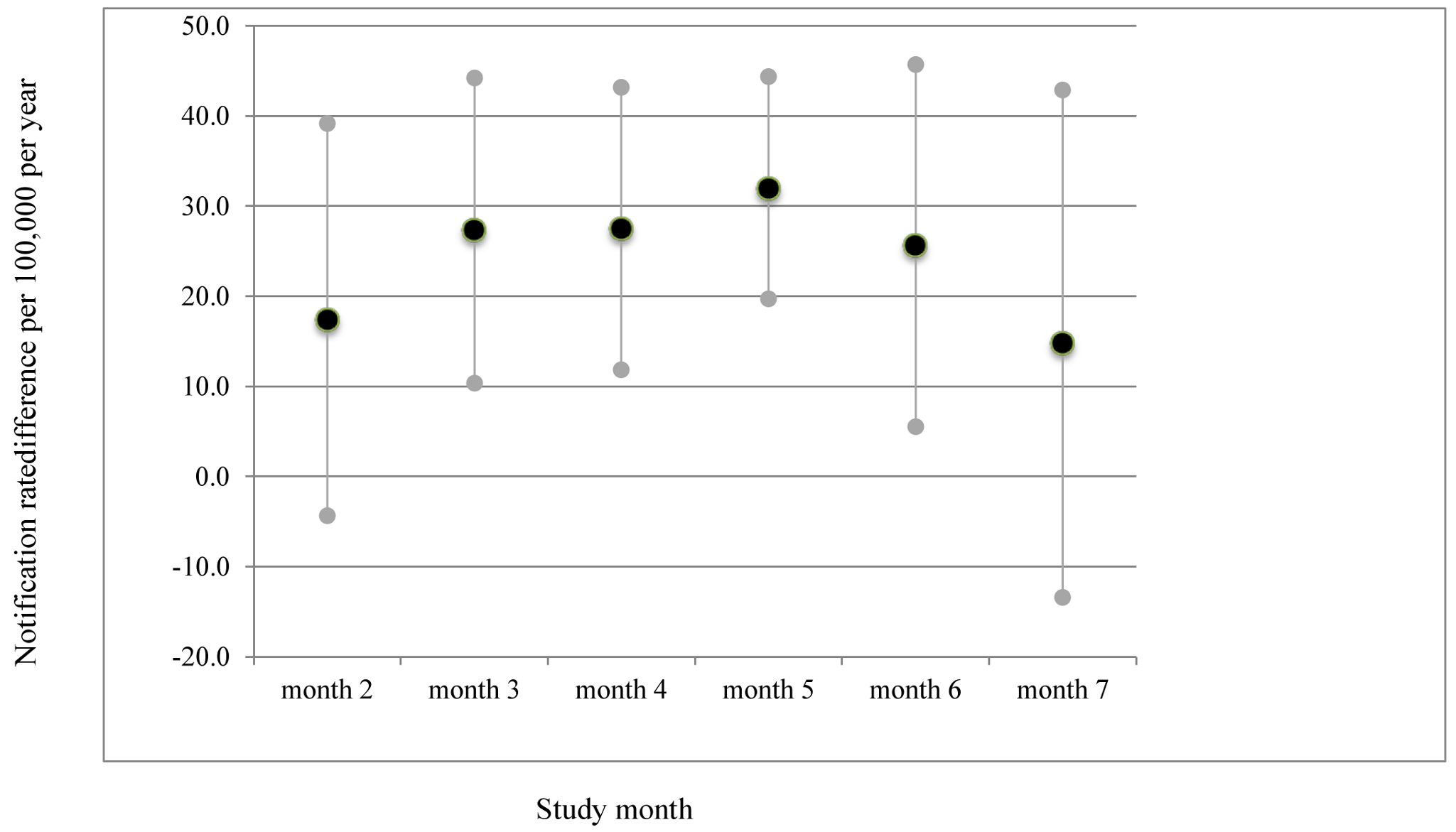

Supplement: Figure S3 — Difference between intervention (Xpert) and baseline (smear examination) arm in cluster-averaged notification rates of laboratory-confirmed TB, by study month. Point estimates represent cluster-averaged notification rate differences between intervention and baseline arms. Values greater than zero denote higher notification rates for intervention than for baseline. Vertical bars: 95% confidence intervals for the cluster-averaged notification rate differences. Horizontal bar: notification rate difference for entire study period (18.1/100,000/year). Month 1 and month 8 had baseline-only and intervention-only observations, respectively. (TIF) [file pmed.1001766.s003.tif]

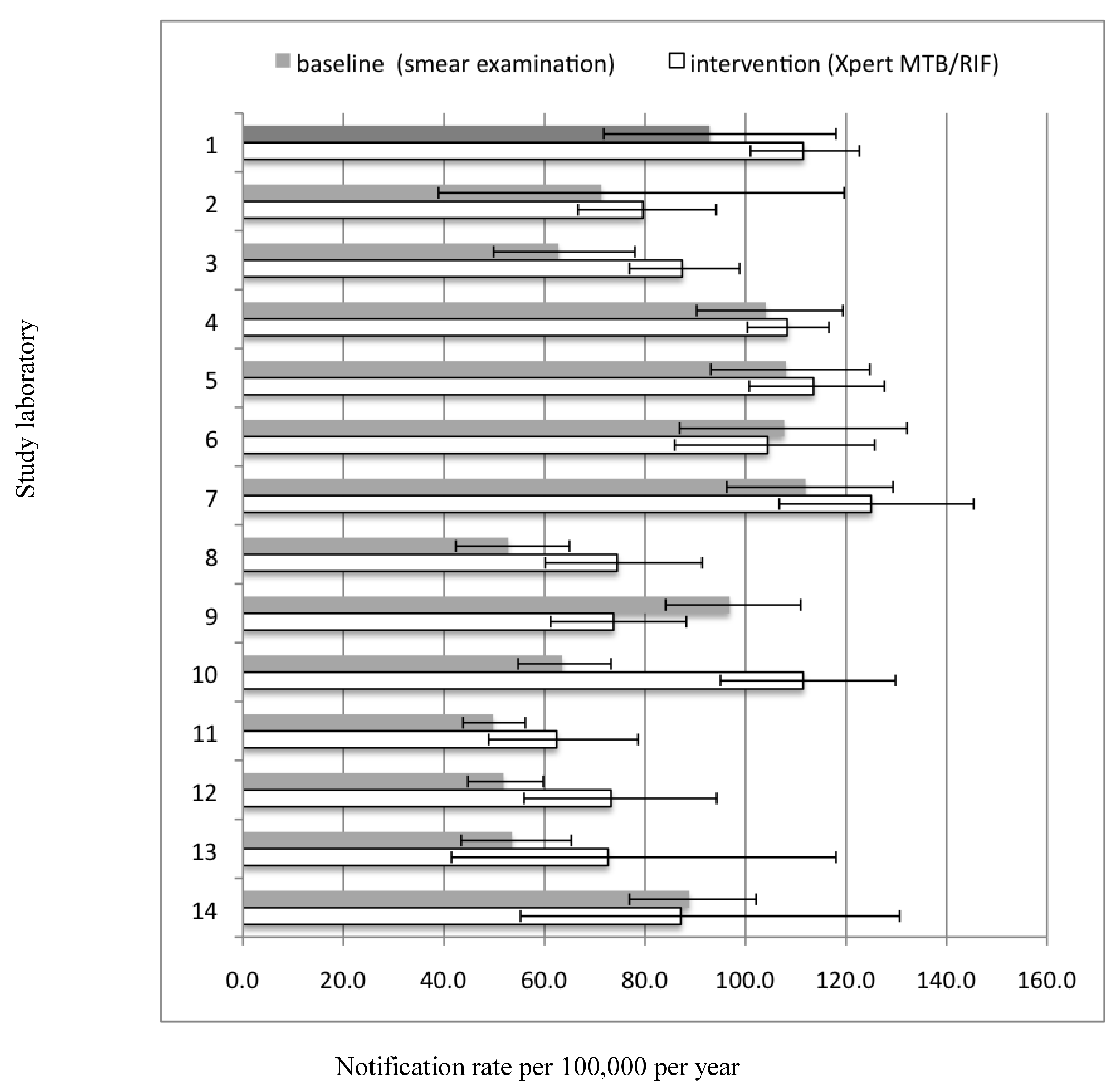

Supplement: Figure S4 — Notification rates for baseline (smear examination) and intervention (Xpert) arms, by study laboratory. Cluster-specific notification rates (i.e., of all clinics that use the services of a particular study laboratory) of overall TB irrespective of laboratory confirmation. Laboratory number corresponds to the sequence of transition from baseline (smear examination) to intervention (Xpert) arm. Laboratories 2, 4, and 6 were situated in Manaus, all others in Rio de Janeiro. (TIF) [file pmed.1001766.s004.tif]

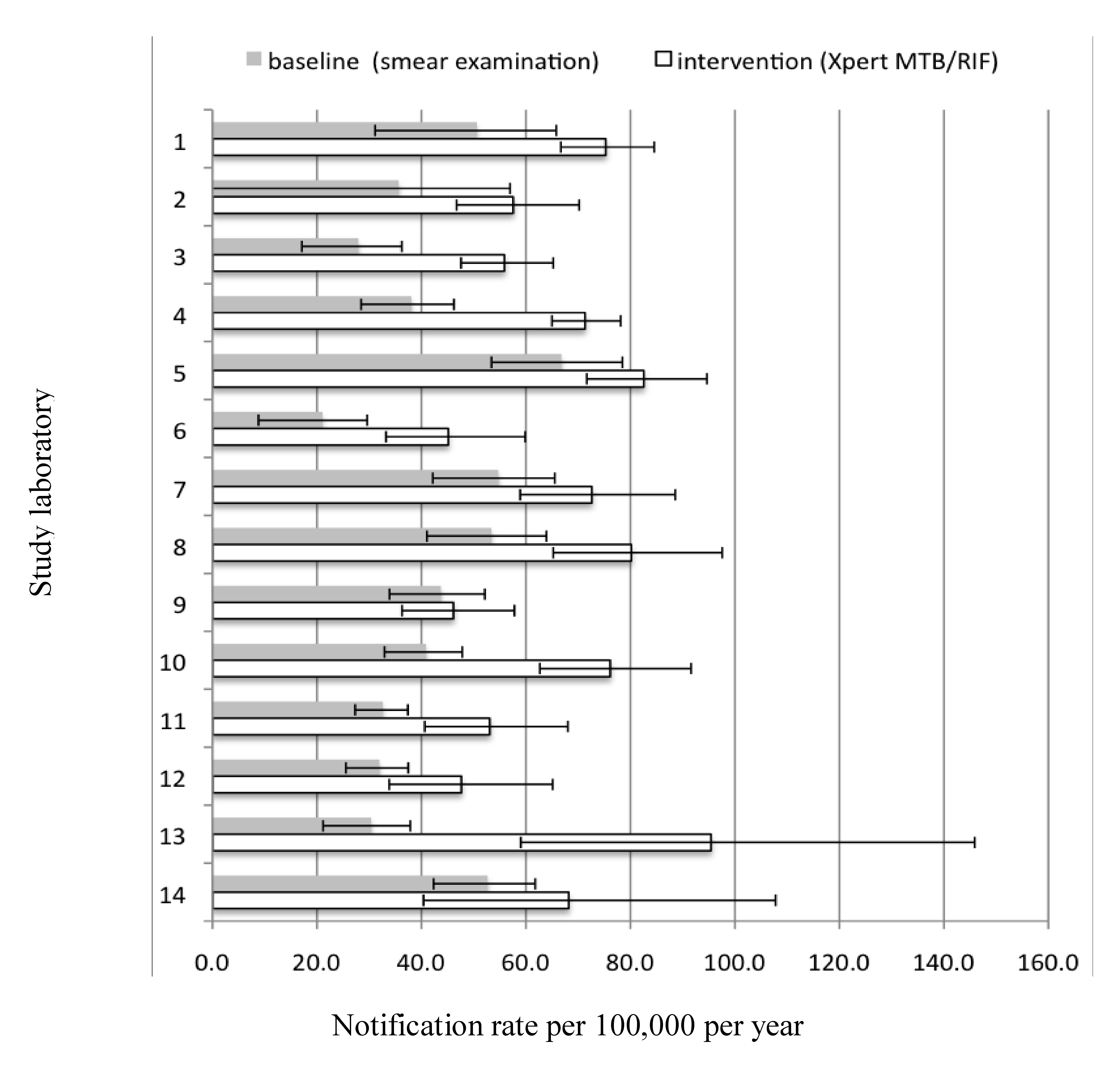

Supplement: Figure S5 — Positivity rate per laboratory, irrespective of notification. Rate per 100,000 population per year for positive laboratory diagnoses, irrespective of notification, per laboratory. Laboratory number corresponds to the sequence of transition from baseline (smear examination) to intervention (Xpert) arm. Laboratories 2, 4, and 6 were situated in Manaus, all others in Rio de Janeiro. (TIF) [file pmed.1001766.s005.tif]
