# Supplementary material for: Impact of Replacing Smear Microscopy with Xpert MTB/RIF for Diagnosing Tuberculosis in Brazil: A Stepped-Wedge Cluster-Randomized Trial
Source: PLoS Med. 2014 Dec 9;11(12):e1001766. doi: 10.1371/journal.pmed.1001766 (PMC4260794; doi:10.1371/journal.pmed.1001766)
Supplement: Table S1 — Numbers and characteristics of laboratory-reported and notified TB cases, by intervention arm, including 54 smear results in the intervention arm (ITT analysis). (DOCX) [file pmed.1001766.s006.docx]

*Table S1 Numbers and characteristics of lab-reported and notified TB cases, by intervention arm, including smear results performed during the intervention arm (Intention-to-treat analysis)*

|  |  | **Baseline arm (smear examination)** | | | | | **Intervention arm (Xpert MTB/RIF)** | | | | | |
| --- | --- | --- | --- | --- | --- | --- | --- | --- | --- | --- | --- | --- |
|  | Number of tests performed | Positive test result | Notified and started on treatment | | | | Number of tests performed | Positive test result | Notified and started on treatment | | | |
|  |  |  | With positive test | Without positive test | | All |  |  | With positive test | Without positive test | | All |
|  |  |  | Positive test result | Negative test result | No test result |  |  |  | Positive test result | Negative test result | No test result |  |
| **Total** ^a^ | 11,705 | 1137 (9.7%) | 831 (40.5%) | 313 (15.3%) | 906 (44.2%) | 2050 (100%) | 14,692 | 1831 (12.5%) | 1439 (54.0%) | 216 (8.1%) | 1009 (37.9%) | 2664 (100%) |
| **Sex** |  |  |  |  |  |  |  |  |  |  |  |  |
| Males | 6,487 | 749 (65.9%) | 555 (66.8) | 202 (64.5%) | 557 (61.5%) | 1314 (64.1%) | 6,679 | 1211 (66.2%) | 940 (65.3%) | 135 (62.5%) | 637 (63.1%) | 1712 (64.3%) |
| Females | 5,218 | 388 (34.1%) | 276 (33.2) | 111 (35.5%) | 349 (38.5%) | 736 (35.9%) | 5,843 | 620 (33.8%) | 499 (34.7%) | 81 (37.5%) | 372 (36.9%) | 952 (35.7%) |
| **Age group** |  |  |  |  |  |  |  |  |  |  |  |  |
| <15 years | 400 | 34 (3.0%) | 25 (3.0%) | 3 (1.0%) | 28 (3.1%) | 56 (2.7%) | 449 | 31 (1.7%) | 21 (1.5%) | 4 (1.9%) | 38 (3.8%) | 63 (2.4%) |
| 15-39 years | 4,786 | 603 (53.0%) | 453 (54.5%) | 165 (52.7%) | 450 (49.7%) | 1068 (52.1%) | 5,057 | 987 (53.9%) | 788 (54.8%) | 113 (52.3%) | 514 (50.9%) | 1415 (53.1%) |
| 40-59 years | 4,228 | 371 (32.7%) | 257 (30.9%) | 108 (34.5%) | 317 (35.0%) | 682 (33.3%) | 4,414 | 618 (33.8%) | 477 (33.1%) | 70 (32.4%) | 310 (30.7%) | 857 (32.2%) |
| ≥60 years | 2,291 | 129 (11.3%) | 96 (11.6%) | 37 (11.8%) | 111 (12.2%) | 244 (11.9%) | 2,602 | 195 (10.6%) | 153 (10.6%) | 29 (13.4%) | 147 (14.6%) | 329 (12.3%) |
| **City** |  |  |  |  |  |  |  |  |  |  |  |  |
| Rio de Janeiro | 9,747 | 1037 (91.2%) | 756 (91.0%) | 228 (72.8%) | 755 (83.3%) | 1739 (84.8%) | 6,989 | 1217 (66.5%) | 911 (63.3%) | 81 (37.5%) | 720 (71.8%) | 1712 (64.3%) |
| Manaus | 1,958 | 100 (8.8%) | 75 (9.0%) | 85 (27.2%) | 151 (16.7%) | 311 (15.2%) | 5,533 | 614 (33.5%) | 528 (36.7%) | 135 (62.5%) | 289 (28.6%) | 952 (35.7%) |
| **HIV status** |  |  |  |  |  |  |  |  |  |  |  |  |
| HIV positive^b^ | N/A | N/A | 54 (6.5%) | 58 (18.5%) | 75 (9.9%) | 187 (9.8%) | N/A | N/A | 88 (6.1%) | 50 (23.1%) | 55 (5.5%) | 193 (7.2%) |
| HIV negative^b^ | N/A | N/A | 284 (34.2%) | 97 (31.0%) | 276 (36.5%) | 657 (34.6%) | N/A | N/A | 382 (26.5%) | 51 (23.6%) | 206 (20.4%) | 639 (24.0%) |
| HIV unknown^b^ | N/A | N/A | 493 (59.3%) | 158 (50.5%) | 405 (53.6%) | 1056 (55.6%) | N/A | N/A | 969 (67.3%) | 115 (53.2%) | 748 (74.1%) | 1832 (68.8%) |
| **TB treatment history** |  |  |  |  |  |  |  |  |  |  |  |  |
| New TB | N/A | N/A | 688 (82.8%) | 266 (85.0%) | 0 | 954 (46.5%) | N/A | N/A | 1229 (85.4%) | 194 (89.8%) | 0 | 1423 (53.4%) |
| Retreatment | N/A | N/A | 142 (17.1%) | 47 (15.0%) | 0 | 189 (9.2%) | N/A | N/A | 210 (14.6%) | 15 (6.9%) | 0 | 225 (8.5%) |
| Unknown | N/A | N/A | 1 (0.1%) | 0 | 906 | 907 (44.2%) | N/A | N/A | 0 | 7 (3.2%) | 1009 | 1016 (38.1%) |

Column percentages, except ^a^. N/A= not available. TB= tuberculosis

^b^excluding 452 notified TB cases not linked to a specified study arm
