# Supplementary material for: Impact of Replacing Smear Microscopy with Xpert MTB/RIF for Diagnosing Tuberculosis in Brazil: A Stepped-Wedge Cluster-Randomized Trial
Source: PLoS Med. 2014 Dec 9;11(12):e1001766. doi: 10.1371/journal.pmed.1001766 (PMC4260794; doi:10.1371/journal.pmed.1001766)
Supplement: Table S2 — Notifications of laboratory-confirmed pulmonary TB by arm (baseline and intervention), by sex, age, municipality, and baseline smear-positive rate, including 54 smear results in the intervention arm (ITT analysis). (DOCX) [file pmed.1001766.s007.docx]

*Table S2 Notifications of laboratory-confirmed pulmonary TB by arm (baseline and intervention), by sex, age, municipality and baseline smear-positive rate, including 54 positive smear results during the intervention arm (Intention-to-treat analysis)*

|  | Baseline arm  (smear examination) | | | Intervention arm  (Xpert MTB/RIF) | | | Notification rate ratio | | Notification rate difference | |
| --- | --- | --- | --- | --- | --- | --- | --- | --- | --- | --- |
|  | Population  (person-years) | Notification rates  (per 100,000 population) | | Population  (person-years) | Notification rates  (per 100,000 population) | |  |  |  |  |
|  |  | overall^b^ | Cluster-averaged^c^  (95% CI) |  | overall^b^ | Cluster-averaged^c^  (95% CI) | overall | Cluster-averaged  (95% CI) | overall | Cluster-averaged  (95% CI) |
| Total | 2799071 | 29.7 | 30.5 (24.9-36.1) | 2647008 | 54.4 | 51.1 (44.0-58.3) | 1.83 | **1.67 (1.39-1.96)** | 24.7 | **20.6 (12.0-29.2)** |
| Sex |  |  |  |  |  |  |  |  |  |  |
| Males | 1465981 | 37.8 | 37.5 (30.7-44.3) | 1404926 | 66.9 | 62.9 (53.8-71.9) | 1.77 | 1.68 (1.39-1.96) | 29.1 | 25.4 (14.6-36.1) |
| Females | 1333090 | 20.7 | 23.1 (17.8-28.5) | 1242082 | 40.2 | 37.7 (30.3-45.1) | 1.94 | 1.63 (1.26-2.01) | 19.5 | 14.6 (5.9-23.4) |
| Age Group |  |  |  |  |  |  |  |  |  |  |
| < 15 years | 594291 | 4.2 | 3.7 (1.2-6.2) | 571447 | 3.7 | 3.1 (1.4-4.7) | 0.87 | 0.83 (0.07-1.59) | - 0.5 | - 0.6 (-3.4-2.2) |
| 15-39 years | 1178082 | 38.4 | 42.5 (31.8-53.2) | 1079807 | 73.0 | 67.5 (56.5-78.5) | 1.90 | 1.59 (1.25-1.93) | 34.6 | 25.1 (10.4-39.7) |
| 40-59 years | 680888 | 37.7 | 36.6 (26.8-46.4) | 640892 | 74.5 | 71.7 (60.7-62.8) | 1.97 | 1.96 (1.57-2.34) | 36.7 | 35.0 (21.0-49.0) |
| >= 60 years | 345811 | 27.7 | 31.3 (19.2-43.3) | 354862 | 43.1 | 44.9 (23.6-66.1) | 1.55 | 1.44 (0.69-2.18) | 15.3 | 13.6 (-0.9-36.9) |
| City |  |  |  |  |  |  |  |  |  |  |
| Rio de Janeiro | 2497035 | 30.3 | 32.1 (25.8-38.6) | 1725565 | 52.8 | 51.6 (42.9-60.2) | 1.74 | 1.61 (1.21-2.31) | 22.5 | 19.5 (9.4-29.6) |
| Manaus | 302036 | 24.8 | 24.6 (1.9-47.1) | 921443 | 57.3 | 49.4 (18.0-80.8) | 2.31 | 2.01 (0.99-3.02) | 32.5 | 24.8 (-0.1-49.8) |
| Baseline rate^a^ |  |  |  |  |  |  |  |  |  |  |
| < 27.5 | 1292644 | 23.5 | 22.3 (15.6-29.0) | 1241463 | 51.7 | 42.8 (28.1-57.5) | 2.20 | 1.92 (1.32-2.53) | 28.2 | 20.6 (7.2-34.1) |
| 27.5-36.4 | 659537 | 29.3 | 29.3 (20.8-37.8) | 471307 | 54.5 | 56.8 (43.9-69.6) | 1.86 | 1.94 (1.53-2.34) | 25.2 | 27.5 (15.6-39.3) |
| ≥ 36.5 | 846890 | 39.5 | 39.8 (30.2-49.4) | 934238 | 57.8 | 54.9 (38.8-71.1) | 1.46 | 1.38 (0.99-1.77) | 18.3 | 15.1 (-0.5-30.8) |

TB= tuberculosis.95% CI= 95% confidence interval.

^a^Lab-specific rate of positive smear examinations in the first study month, per 100,000 population per year

^b^Overall notification rates: number of notified cases divided by population size, multiplied by 100,000.

^c^Cluster-averaged rates: mean of cluster-specific notification rates.
