# Supplementary material for: Impact of Replacing Smear Microscopy with Xpert MTB/RIF for Diagnosing Tuberculosis in Brazil: A Stepped-Wedge Cluster-Randomized Trial
Source: PLoS Med. 2014 Dec 9;11(12):e1001766. doi: 10.1371/journal.pmed.1001766 (PMC4260794; doi:10.1371/journal.pmed.1001766)
Supplement: Table S3 — Secondary analysis: unadjusted and multivariably adjusted notification rate ratios for laboratory-confirmed TB, TB with negative test result, TB with no testing, and overall pulmonary TB, using a mixed multilevel model. (DOCX) [file pmed.1001766.s008.docx]

**Table S3 Secondary analysis: unadjusted and multivariably adjusted notification rate ratios for laboratory-confirmed TB, TB with negative test result, TB with no testing, and overall pulmonary TB, using a mixed multilevel model^a^**

|  | Mixed multilevel model^a^ | | | | | | | | | |
| --- | --- | --- | --- | --- | --- | --- | --- | --- | --- | --- |
|  | Unadjsuted | | Adjusted^b^ | | | Unadjusted | | | Time-adjusted^c^ | |
|  | NRR (95% CI) | P-value | | NRR (95% CI) | P-value | | NRR (95% CI) | P-value | NRR (95% CI) | P-value |
| Laboratory-confirmed notifications | 1.59 (1.31-1.88) | <0.001 | | 1.59 (1.32-1.87) | <0.001 | | 1.59 (1.44-1.76) | <0.001 | 1.70 (1.51-1.92) | <0.001 |
| Notifications despite negative lab result | 0.61 (<0.01-1.23) | 0.206 | | 0.52 (0.21-0.84) | 0.004 | | 0.63 (0.52-0.77) | <0.001 | 0.66 (0.49-0.90) | 0.008 |
| Notifications with no lab test requested | 0.97 (0.63-1.30) | 0.850 | | 0.98 (0.64-1.32) | 0.923 | | 1.00 (0.90-1.11) | 0.948 | 1.20 (1.03-1.40) | 0.022 |
| All notifications | 1.15 (0.94-1.37) | 0.157 | | 1.16 (0.96-1.37) | 0.115 | | 1.18 (1.10-1.26) | <0.001 | 1.30 (1.18-1.44) | <0.001 |
| Positive laboratory examinations | 1.60 (1.33-1.86) | <0.001 | | 1.62 (1.40-1.84) | <0.001 | | 1.58 (1.45-1.73) | <0.001 | 1.47 (1.33-1.64) | <0.001 |

NRR= notification rate ratio for intervention (Xpert MTB/RIF) compared to baseline (smear examination) arm. 95% CI= 95% confidence interval. TB= tuberculosis

^a^ laboratory specified as the first level

^b^ adjusted for sex, age, municipality and baseline smear-positive rate, quasi-likelihood population-averaged method

^c^ adjusted for sex, age, municipality, baseline smear-positive rate and calendar time (2-month blocks)
