# Supplementary material for: Impact of Replacing Smear Microscopy with Xpert MTB/RIF for Diagnosing Tuberculosis in Brazil: A Stepped-Wedge Cluster-Randomized Trial
Source: PLoS Med. 2014 Dec 9;11(12):e1001766. doi: 10.1371/journal.pmed.1001766 (PMC4260794; doi:10.1371/journal.pmed.1001766)
Supplement: Table S4 — Notification rate ratios of laboratory-confirmed TB adjusted for calendar time, comparing the Xpert to the smear microscopy arm, stratified by baseline smear-positive rate (time-adjusted mixed multilevel model). The time-adjusted mixed multilevel model for laboratory-confirmed notifications showed significant interactions between intervention status and municipality, and between intervention status and baseline smear positivity rate. The interaction with municipality no longer contributed significantly to the model likelihood when the interaction with baseline rate was included in the model, whereas the interaction with baseline rate continued to contribute significantly (p<0.001) even when the interaction with municipality was included. Hence, we concluded that the underlying interaction was between intervention status and baseline smear positivity rate. This table shows that the notification rate ratios for laboratory-confirmed TB decreased from 1.97 in the lowest baseline category to 1.28 in the highest baseline category. (DOCX) [file pmed.1001766.s009.docx]

**Table S4 Notification rate ratios of laboratory-confirmed TB adjusted for calendar time, comparing the Xpert to the smear arm, stratified by baseline smear-positive rate (time-adjusted mixed multilevel model).**

| **Baseline smear-positive rate** | NRR (95% CI) | P-value |
| --- | --- | --- |
| <27.5/100,000/year | 1.97 (1.58-2.46) | <0.001 |
| 27.5-36.4/100,000/year | 1.79 (1.33-2.42) | <0.001 |
| ≥ 36.5/100,000/year | 1.28 (1.01-1.61) | 0.04 |

NRR=notification rate ratio

95% CI= 95% confidence interval
