# Supplementary material for: Impact of Replacing Smear Microscopy with Xpert MTB/RIF for Diagnosing Tuberculosis in Brazil: A Stepped-Wedge Cluster-Randomized Trial
Source: PLoS Med. 2014 Dec 9;11(12):e1001766. doi: 10.1371/journal.pmed.1001766 (PMC4260794; doi:10.1371/journal.pmed.1001766)
Supplement: Table S5 — Cluster-averaged analysis excluding month 1 and month 8. Excluding the data for months 1 and 8, which related to baseline-only and intervention-only observations, respectively, did not affect the cluster-averaged notification rate ratio for laboratory-confirmed TB (1.60, 95% CI 1.25, 1.96, p<0.01), although the notification rate ratio adjusted by quasi-likelihood population-averaged analysis was lower (1.48, 95% CI 1.17, 1.79, p<0.01). These exclusions slightly increased the unadjusted and adjusted cluster-averaged notification rate ratios for overall TB. (DOCX) [file pmed.1001766.s010.docx]

**Table S5 Univariate and multivariate notification rate ratios for laboratory-confirmed TB and overall TB, after excluding study months 1 and 8, by analysis method.**

|  | **cluster-averaged** | | | | | | **mixed multilevel model^a^** | | | | | |
| --- | --- | --- | --- | --- | --- | --- | --- | --- | --- | --- | --- | --- |
|  | unadjusted | | | adjusted^b^ | | | unadjusted | | | time-adjusted^c^ | | |
|  | NRR | 95% CI | P-value | NRR | 95% CI | P-value | NRR | 95% CI | P-value | NRR | 95% CI | P-value |
| Laboratory-confirmed notifications | 1.60 | (1.25-1.96) | 0.002 | 1.48 | (1.17-1.79) | <0.01 | 1.61 | (1.42-1.84) | <0.001 | 1.76 | (1.55-1.99) | <0.001 |
| All notifications | 1.27 | (0.98-1.55) | 0.063 | 1.22 | (0.98-1.46) | 0.08 | 1.28 | (1.18-1.40) | <0.001 | 1.41 | (1.30-1.54) | <0.001 |

NRR = notification rate ratio for intervention compared to baseline period; 95% CI= 95% confidence interval

^a^laboratory specified as the first level

^b^adjusted for sex, age, municipality and baseline smear-positive rate, quasi-likelihood population-averaged method

^c^adjusted for sex, age, municipality, baseline smear-positive rate and calendar time (2-month blocks)
