# Supplementary material for: Impact of Replacing Smear Microscopy with Xpert MTB/RIF for Diagnosing Tuberculosis in Brazil: A Stepped-Wedge Cluster-Randomized Trial
Source: PLoS Med. 2014 Dec 9;11(12):e1001766. doi: 10.1371/journal.pmed.1001766 (PMC4260794; doi:10.1371/journal.pmed.1001766)
Supplement: Text S1 — Trial protocol. (DOC) [file pmed.1001766.s011.doc]

**Study Protocol**

**Pilot roll out for GeneXpert for the diagnosis of pulmonary tuberculosis in two municipalities in Brazil**

*Principal investigator*:

Dr. Betina Durovni, Special Advisor, Municipality of Rio de Janeiro Health Department, Brazil

*Co-principal investigator*:

Prof. Reynaldo Dietze, Federal **University** of Espírito Santo, Vitória, Brazil

*Collaborating institutions*:

National Tuberculosis Control Program, Ministry of Health, Brasilia, Brazil

Ataulpho de Paiva Foundation, Rio de Janeiro, Brazil

Oswaldo Cruz Foundation, FIOCRUZ, Rio de Janeiro, Brazil

Tuberculosis Research Network, REDE-TB, Brazil

Bill and Melinda Gates Foundation, Seattle WA, USA

Center for Poverty-related Communicable Diseases - Academic Medical Center, Amsterdam, Netherlands

KNCV Tuberculosis Foundation, The Hague, Netherlands

Project identifier

|  | **Country: Brazil** | **Funding:**  **Bill & Melinda Gates Foundation** |
| --- | --- | --- |
| **Planning Period:**  **1 March 2011 – 30 September 2012** |  | |

**Abbreviations**

CI Confidence interval

DEFF Design effect

DEFF_SW_ Design effect due to stepped-wedge design

DISA District (Manaus)

DST Drug susceptibility testing

FIND Foundation for innovative new diagnostics

FMATM Fundação de Medicina Tropical do Amazonas

GX GeneXpert® MTB/RIF

LACEN Laboratório Central Noel Nutels

MDR Multidrug resistance

MoH Ministry of Health

NTCP National Tuberculosis Control Program

PCF Cardoso Fontes Policlinic

PCR Polymerase chain reaction

PCU Primary Care Unit

PLHA Person living with HIV/aids

QA Quality assurance

SINAN Sistema de Informação de Agravos de Notificação (notification system)

SMS Secretaria Municipal de Saúde (Municipal Health Secretariat)

SOP Standard operating procedure

TB Tuberculosis

WHO World Health Organization

**Contents**

**I. INTRODUCTION** [4](#__RefHeading___Toc293985492)

**II. STUDY OBJECTIVES** [6](#__RefHeading___Toc293985493)

1. Aim [6](#__RefHeading___Toc293985494)

2. Objectives [6](#__RefHeading___Toc293985495)

3. Expected outcomes [6](#__RefHeading___Toc293985496)

**III. STUDY POPULATION AND DESIGN** [7](#__RefHeading___Toc293985497)

1. Study population [7](#__RefHeading___Toc293985498)

2. Study design [8](#__RefHeading___Toc293985499)

3. Intervention [9](#__RefHeading___Toc293985500)

4. Study endpoints [10](#__RefHeading___Toc293985501)

5. Sample size [10](#__RefHeading___Toc293985502)

6. Study duration and phasing [12](#__RefHeading___Toc293985503)

7. In- and exclusion criteria [12](#__RefHeading___Toc293985504)

8. Data collection [13](#__RefHeading___Toc293985505)

9. Laboratory methods [13](#__RefHeading___Toc293985506)

10. Data management and analysis [13](#__RefHeading___Toc293985507)

11. Sources of bias [15](#__RefHeading___Toc293985508)

12. Quality assurance [15](#__RefHeading___Toc293985509)

**IV. ETHICAL ISSUES** [16](#__RefHeading___Toc293985510)

**V. RESEARCH TEAM** [17](#__RefHeading___Toc293985511)

**VI. LIST OF STANDARD OPERATING PROCEDURES AND FORMS** [17](#__RefHeading___Toc293985512)

**IX. BUDGET (in US$)** [18](#__RefHeading___Toc293985513)

**X. REFERENCES** [18](#__RefHeading___Toc293985514)

# **I. INTRODUCTION**

Tuberculosis (TB), a preventable and curable disease, persists through the centuries as public health problem worldwide and is an important cause of morbidity and mortality, mainly in developing countries. Despite the existence, for more than 50 years, of efficient antituberculous drugs and an effective treatment to prevent progression of latent TB to active disease, TB is among the ten leading causes of death in the world.[[1]](#endnote-2) In 2009, 9.4 million people had TB and 1.7 million died with TB in the world, of whom 0.5 million living with HIV/aids (PLHA). This represents 4700 daily deaths from TB, which is responsible for ¼ of premature deaths. Among TB, TB causes more deaths than breast cancer and maternity-related conditions.[[2]](#endnote-3)

With these figures, it is not surprising that the United Nations have included among the 8 Millennium Development Goals the control of TB (and other infectious diseases). The goal is to eradicate TB until 2050, decreasing by half the incidence rates.[[3]](#endnote-4) In order to achieve these objectives, the *Union* (International Union Against Tuberculosis and Lung Diseases) proposed, in 1995, the directly observed treatment strategy (DOTS). During one decade, the World Health organization (WHO) widely promoted this strategy and estimated that 8 million deaths were averted. However, in 2005, WHO recognized that the strategy would not be sufficient to attain the millennium goals, and proposed a more comprehensive strategy, the Stop TB Partnership. In a recent review of the intermediate objectives for the 2011-2015 period, the goal was set to detect at least 84% of smear-positive cases and to cure at least 87% of them.Error: Reference source not found

Indeed, the early detection of smear-positive cases and the immediate start of therapy are considered as the most important actions for TB control. Treatment reduces rapidly the number of infectious organisms transmitted by patients thus preventing transmission and the occurrence of secondary cases. In other words, it reduces the number of new patients with TB in the community (incidence) through the cure of prevalent cases. The correct treatment also reduces morbidity, TB-related mortality and drug-resistance.

According to WHO, Brazil has the highest number of TB cases in Latin America and is among the 22 high burden countries, a priority for the disease control, since they concentrate 80% of all TB cases in the world.Error: Reference source not found According to the Brazilian National Information System (SINAN) from the Ministry of Health (MoH), in 2009 72,000 cases were notified, out of the 93,000 estimated by WHO, and 4,700 TB-related deaths occurred in the country. Rio de Janeiro and Amazonas states present the highest incidence rates, 97 and 82 cases per 100,000 inhabitants respectively, corresponding to the double of the national incidence rate. The cities of Rio de Janeiro and Manaus, capitals of Rio de Janeiro and Amazonas states occupy the second and fifth places in the ranking of the highest incidence rates in the country.[[4]](#endnote-5)

The available tuberculosis (TB) diagnostic tools have several limitations: sputum smear examination can be done at the lowest health care levels but has a low sensitivity and therefore two smears usually are examined. Culture is more sensitive and may confirm TB in those with negative smears. Also drug susceptibility testing (DST) requires culturing first. However, culturing and especially DST have a long turnaround time and a higher level of biosafety is required. Rapid PCR methods are available but they require a specialized infrastructure.

In Brazil, smear examinations are widely available for patients with TB suspicion, free of charg for patients, in public health units. Culture is only recommended by the National TB Control Program (NTCP) in patients with previous history of TB, in PLHA, when treatment failure is suspected by the end of the second month of treatment, in difficult to obtain specimens, in extrapulmonary TB and in contacts of a resistant TB index case. Since sputum smear examination has a low sensitivity, the bulk of smear-negative TB diagnoses is made clinically without microbiological confirmation.[[5]](#endnote-6) In addition, despite the official recommendations, according to data from the NTCP, only 72% of new cases and only 17% of retreatment cases perform culture.Error: Reference source not found If added to cases with a negative sputum smear, at present, only one third of Brazil’s notified new cases are bacteriologically confirmed. It is estimated that 20% of these cases are not true TB cases (false positive diagnosis), resulting in delayed diagnosis of the true disease and unnecessary exposure to hepatotoxic antituberculous drugs. In conclusion, the limitations of the currently available tests for TB result in delayed diagnosis and unnecessary treatment.[[6]](#endnote-7),[[7]](#endnote-8)

In order to increase the detection of sensitive TB and to accelerate the diagnosis of resistant TB, the NTCP had the plan, in 2010, to implement universal culture (for every patient suspect of TB). Nevertheless, at present, the national laboratories that perform culture and DST, the state LACENs (Laboratório Central Noel Nutels) cannot absorb this workload, a great investment would be necessary.

A novel test platform has been developed, which is based on rapid real-time PCR. The all-in-one cartridge method allows use outside specialized laboratories. A version for detection of *M. tuberculosis*, the GeneXpert, has been developed with funding by FIND, the Foundation for Innovative New Diagnostics. Controlled trial in over 1700 TB suspects showed a sensitivity of 92.2%, representing a significant gain vis-à-vis sputum smears. In sputum smear negative samples, the sensitivity of GeneXpert® is 72.5% in one sample and reaches 90.2% in three samples.[[8]](#endnote-9)-[[9]](#endnote-10)[[10]](#endnote-11)[[11]](#endnote-12)[[12]](#endnote-13)[[13]](#endnote-14)[[14]](#endnote-15)[[15]](#endnote-16)[[16]](#endnote-17)[[17]](#endnote-18)[[18]](#endnote-19)[[19]](#endnote-20)

This assay also contains primers for the core region of the *rpoB* gene which contains more than 95% of the gene mutations responsible for resistance to rifampicin. Most (≥95%) strains resistant to rifampicin also are resistant to isoniazid.[[20]](#endnote-21) These with multidrug resistant (MDR) TB strains need second-line drugs to be successfully treated. Therefore, rapid detection of rifampicin resistance is important for the patient and prevention of transmission of MDR-TB. This GeneXpert assay has the potential to revolutionize the TB diagnostic process: with its high sensitivity and specificity it can replace smear examination and culture while being applicable in a routine laboratory with minimum infrastructure and limited training levels of staff.

GeneXpert® MTB/RIF is already available in private laboratories in Brazil since 2009, when it was approved by the national regulatory agency ANVISA, equivalent to the USA FDA (DOU supplement Nº 204, Monday, 26 October 2009 - ISSN 1677-7042, attached). Despite the above mentioned evidence of accuracy and potential to reduce diagnosis delay and TB transmission, the Brazilian Unified Health System (SUS) has not yet incorporated this technology, which deprives most of the TB population from its access.

The Government of Brazil and the Bill and Melinda Gates Foundation have entered a high-level agreement for transforming diagnosis and treatment of TB in Brazil. The GeneXpert assay will be validated against culture in Brazil in a study sponsored by FIND. As a complement to the validation protocol, co-developed by FIOCRUZ and FIND and planned to begin in March 2011 (approved by CONEP 630/2010), a study to evaluate cost-effectiveness and potential rollout strategies for GeneXpert assay are presently being proposed in two different protocols. National cost-effectiveness data is required by the Committee for Incorporation of Technologies in Health (CITEC) from the Ministry of Health to allow for the adoption of the GeneXpert assay, providing a basis for evidence-driven policy in this area. This requirement intends to assure that the benefits of incorporating any new technology into the public health system will outweigh the costs needed to make this a reality, i.e., that it has a significant impact on public health at an affordable and justifiable cost. While the validation study will show the potential of the assay, its cost-effectiveness in comparison to existing methods, its size, scope and generalizability will be limited as it will look at test performance and impact on treatment decisions in a limited number of settings.

In order to really demonstrate how the assay should be incorporated in the Brazilian health system, and what level of impact on TB case finding and early appropriate treatment can be expected when applied on a large scale, an expanded pilot introduction is needed. This planned roll-out proposed in the present protocol will also offer the opportunity to complement the planned cost-effectiveness analyses with potential cost savings outside the health system, in particular by incorporating patients’ opportunity costs (separate protocols will be submitted for ethical approval). We therefore propose to assess the potential application for GeneXpert in the Brazilian Health System context as part of a pilot roll out, using a “programmatic cohort” approach that can assess impact. This pilot roll out will be closely monitored and evaluated to document added value and inform broader adoption and roll out in other cities in Brazil.

As it will be afore detailed, the present rollout study proposes a change in the laboratory routine, not in the primary care health units (PCU). Randomization and data analysis will not be individual, but by laboratory/PCU. There is consistent evidence that this change will bring benefits to patients and their contacts, since an early bacteriologically confirmed diagnosis is foreseen, with consequent reduction in transmission. It is expected that this study will provide the NTCP with information for decision making regarding implementation of the technology, necessary personnel training, impact of the test in PCU and laboratory routines and other operational information.

In addition to the increased detection rate, secondary benefits from the substitution of GeneXpert for smears are expected, such as reduction of diagnosis delay of sensitive and drug-resistant TB, of morbidity, mortality and transmission. The reduction in the time to diagnosis of MDR-TB will allow the early treatment with second-line drugs recommended in these cases, also reducing morbidity and mortality, offering less opportunity for MDR-TB transmission. Finally, a reduction in the number of cases erroneously treated as TB is expected. Thus, this project ultimately intends to contribute to TB control in the country.

We emphasize that the present protocol includes only the operational pilot implementation study, other protocol submissions will follow for cost-effectiveness and acceptability studies. The validation study, previously submitted, was approved by CONEP and should be started in March 2011.

# **II. STUDY OBJECTIVES**

## 1. Aim

To improve tuberculosis control in Brazil by increasing the effectiveness of the diagnostic process of pulmonary TB through introduction of novel diagnostic tools.

## 2. Objectives

To estimate, in the routine of public health facilities of two municipalities in Brazil, the impact of the implementation of the GeneXpert assay on

- the case detection of pulmonary TB, measured by the change in rate of notification and treatment registration of bacteriologically confirmed TB, after introduction of the GeneXpert assay as a replacement of sputum smear microscopy in the routine health care settings.
- the detection of MDR-TB

## 3. Expected outcomes

It is expected that the project will provide recommendations to the NTCP regarding changes in the testing algorithm and the potential impact, to inform the sequence of rollout in other states and cities, type of service in which GeneXpert could be used, coverage, acceptability, etc.

The project will support planning, coordination, capacity building, monitoring, equipment and the required supplies for the pilot introduction.

# **III. STUDY POPULATION AND DESIGN**

## 1. Study population

The study will look at the *health system* rather than at individual clinics, i.e. will in both municipalities include a diagnostic center together with all clinics that drain on it. The reason is that introduction of new diagnostics may lead to changes in referral which the study should capture.

In **Rio de Janeiro** there are 10 health districts belonging to the municipal network, each with at least one facility with TB smear examination capacity that serves the other health facilities in the district. If sputum smear examination is requested by the clinic, two specimens are transported to the district microscopy facility for examination. Culture is performed in 4 municipal reference labs: P. Hélio Pellegrino, P. Carlos Alberto Nascimento, H. Municipal Rafael de Paula Souza e H. N. Sra do Loreto. DST is performed in four specialized reference labs that operate outside the Municipal network: Federal University Hospital, Hélio Fraga TB Reference Center, State Lab and Fiocruz. In addition TB is diagnosed in a number of general and emergency hospitals.

Rio de Janeiro has  an estimated population of 6,291,773  in 2008 , and a total TB notification of 7,349 cases among people living in the city (overall notification rate 2008 of 116.8/100,000), including 3,401 Smear-positive pulmonary cases (notification rate 54.1 /100,000), 1,614 smear-negative pulmonary cases (notification rate 25.7/100,000), and 910 extrapulmonary cases (notification rate 14.5/100,000). Of all reported TB cases, 65% are diagnosed by primary care units.[[21]](#endnote-22) In Rio de Janeiro, all 11 laboratories will use the new technology for diagnosisng TB, as detailed in the Methods section.

In **Manaus** there are several basic (primary) care units with TB smear microscopy capacity. The city network is divided into 4 areas, each with a main lab that handles TB diagnostics for the area, but with large variation in numbers of specimens examined and yet limited coordination. Other important diagnostic centers are the Policlinic Cardoso Fontes (PCF) and de Fundação de Medicina Tropical do Amazonas (FMTAM). PCM is the main TB diagnostic center in the city and receives most referrals for TB diagnosis and treatment; it detects 50% of the TB cases in Manaus. FMTAM is an HIV in- and outpatient clinic that handles most TB-HIV co-infection cases. TB culture is done at two sites: LACEN-AM and FMTAM. Manaus in 2008 had an estimated population of 1,73 million, and a smear-positive total TB notification of 1,775: 1,480 pulmonary and 295 extrapulmonary cases (overall notification rate 94/100,000), including 957 (64.7%) smear-positive pulmonary cases 427 smear-negative pulmonary cases (28.9%), and 96 cases in whom no smear test was performed.

During the roll out pilot, the GeneXpert assay will be introduced at each of the 11 laboratories that provide sputum smear microscopy in Rio de Janeiro: this will be the most logical and feasible location for a diagnostic procedure that is to replace smear examination. Since there is no overlap between the health districts in terms of smear microscopy services, this also provides clear separation between allocation units (see Study design, below). This will make calculation of rates based on population denominators straightforward.

In Manaus the GeneXpert assay will be introduced at 3 sites: PCM, FMTAM and one of the 4 TB labs in the city network: the Basic Health Care unit of the Eastern District (Disa Leste). These three sites together take approximately 70% of the current smear examination load in the city. In order to limit overlap in catchment population between these sites and increase coverage of the entire city population, primary care clinics will as much as possible divert, for the duration of the pilot project, their smear examination requests to one of these three sites.

| 2. Study design The design will be a group-based comparison with phased introduction of the GeneXpert (*stepped-wedge* or *phased implementation* design).[[22]](#endnote-23) The units of comparison will be defined as smear examination laboratories together with the clinics that drain upon them for this laboratory service, as defined above. A group-wise comparison is preferred because the intervention will be introduced at the group level (i.e. in laboratories) and the impact of the intervention is thus best assessed at the group level as well. Also, designs with individual comparisons (e.g. individual randomization) will disrupt clinic and laboratory routine and therefore introduce artificial elements that may bias the outcomes (Hawthorne effect).  In this design, all units will start in the *baseline* group, i.e. diagnose TB following current procedures. Each unit will then at different points in time change to the *intervention* group i.e. do GeneXpert instead of smear examination, such that at the end of the observation period all units will be in the intervention group. The sequence by which the units move from baseline to intervention will be randomized. In that way all units will contribute to the person-time observation of both the baseline and the intervention group, but with different durations. This will allow before-after comparison while taking account of potential bias that would be introduced if e.g. case detection rates change over time for reasons not related to the intervention.  See figure 1 for graphic explanation of the design.  *Figure 1. Graphic representation of study design.* | | | | | | | | | |
| --- | --- | --- | --- | --- | --- | --- | --- | --- | --- |
| **Time** | month 1 | month 2 | month 3 | month 4 | month 5 | month 6 | month 7 | month 8 |  |
| **Unit** |  |  |  |  |  |  |  |  |  |
| 1 |  |  |  |  |  |  |  |  |  |
| 2 |  |  |  |  |  |  |  |  |  |
| 3 |  |  |  |  |  |  |  |  |  |
| 4 |  |  |  |  |  |  |  |  |  |
| 5 |  |  |  |  |  |  |  |  |  |
| 6 |  |  |  |  |  |  |  |  |  |
| 7 |  |  |  |  |  |  |  |  |  |
| 8 |  |  |  |  |  |  |  |  |  |
| 9 |  |  |  |  |  |  |  |  |  |
| 10 |  |  |  |  |  |  |  |  |  |
| 11 |  |  |  |  |  |  |  |  |  |
| 12 |  |  |  |  |  |  |  |  |  |
| 13 |  |  |  |  |  |  |  |  |  |
| 14 |  |  |  |  |  |  |  |  |  |
|  | | | | | | | | | |
|  |  | baseline | |  | intervention | |  |  |  |

*Randomization procedure*

Before the start of the study all units will be listed, assigned a random number between 0 and 13 using a random number generator (uniform distribution) and ranked according to the random number’s sequence.

Due to the relatively small number of units, imbalanced randomization with respect to important (confounding) variables is well possible despite randomization. Therefore, we will apply restrained randomization based at least on size of the case load of the laboratories and the estimated HIV prevalence among the patients using the method proposed by Moulton et al.

## 3. Intervention

The intervention will be the introduction of the GeneXpert assay as replacement of sputum smear microscopy in routine health care settings.

During the **baseline period** all patients suspected clinically of pulmonary TB will have sputum smear examination as per routine and current NTCP guidelines, i.e. two sputum samples will be sent with a request form to the microscopy facility. The result is then communicated back to the clinic. In case of one or two positive smear examinations the patient is diagnosed with smear-positive TB and treated accordingly. In case of two negative smears the diagnosis of smear-negative TB is made or rejected on clinical grounds, generally including chest X-ray examination and an assessment of the clinical response to broad-spectrum antibiotics. Repeat smear examination may be done, as may be mycobacterial culture in case the patient qualifies the NTCP selection criteria. Since culture results may take 4 to 8 weeks, the patient may be initiated on treatment before the result becomes available if the clinical condition so requires. A negative culture result then may or may not result in discontinuation of TB treatment.

During the **intervention period** all patients suspected clinically of pulmonary TB will have sputum examination as per routine and current NTCP guidelines, but instead of microscopic smear examination of two specimens, one sputum specimen will be submitted examined using the GeneXpert (Figure 2).The result is then communicated back to the clinic. In case of a positive result the patient is diagnosed with pulmonary TB and treated accordingly. In addition, microscopic sputum smear examination will be done to classify the patient as smear-positive or smear-negative for notification purposes. Note that this will *only be done in case of a positive GeneXpert result* (i.e. for the minority of TB suspects). In case of a positive Genexpert but a negative smear examination, a second sputum specimen will be requested before treatment initiation and examined microscopically in order to fulfill the notification criteria for smear-negative TB (i.e. TB with at least two negative smears).

In case of a negative GeneXpert result but continued clinical suspicion of TB the GeneXpert can be repeated after a time period determined by the clinical condition of the patient.

In case of a positive GeneXpert result with a positive signal for rifampicin resistance the GeneXpert assay will be repeated on a different sputum specimen. If a mutation in the rifampicin resistance-conferring gene is confirmed, the patient will be referred for further evaluation and treatment of MDR-TB as per routine and NTCP guidelines. See for this also the section on ethical considerations. Patients with a confirmed signal for rifampin resistance will be referred to the MDR referral center for further evaluation including culture and DST.

Figure 2: flow chart of laboratorial procedures.


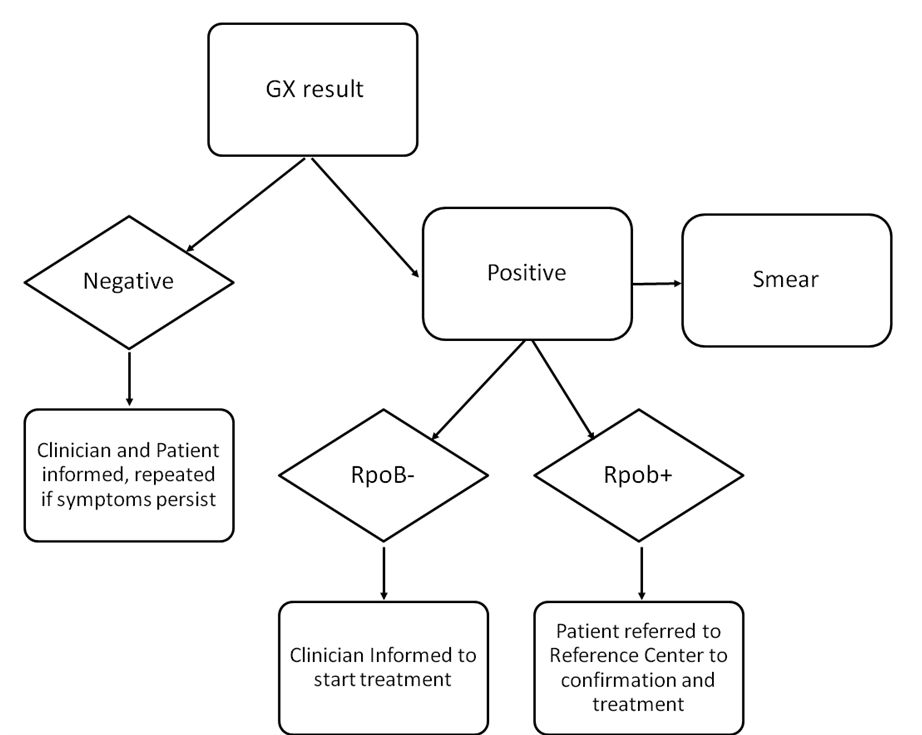


## 4. Study endpoints

The study will have the following endpoints:

1. The change in incidence rates of diagnosed and notified bacteriologically confirmed pulmonary TB comparing the baseline to the intervention period, expressed per 100,000 population per annum. We will take the rate of diagnosed TB as well as that of notified TB since the latter is restricted to patients who are put on treatment within the public health system, and data show that 10-15% of the diagnosed cases are not notified. Bacteriological confirmation will be defined as: a positive microscopic smear examination *or* a positive culture with identification positive for *M. tuberculosis,* *or* a positive MTB signal in the GeneXpert assay.
2. The change in incidence rates of diagnosed, but not bacteriologically confirmed, pulmonary TB comparing the baseline to the intervention period, expressed per 100,000 population per annum.
3. The change in incidence rates of diagnosed and notified bacteriologically confirmed (by the GeneXpert) smear-negative pulmonary TB comparing the baseline to the intervention period, expressed per 100,000 population per annum. This is the measure in which the greatest impact is expected.
4. The change in proportion of diagnosed pulmonary TB patients who are ultimately diagnosed as having MDR-TB, comparing patients enrolled in the baseline period with patients enrolled in the intervention period.

## 5. Sample size

Since the study will done in two preselected municipalities and the distribution and number of TB microscopy services in these municipalities is largely predefined by the routine health care conditions under which we wish to observe the impact of a new diagnostic tool, the number of units for this study is fixed at 14. Since also the expected baseline TB notification rate for these units is fixed, the effective sample size can only be varied by varying the observation period.

Sample size calculations were based on the following principles and assumptions:

- Units serve a population of on average 500,000 inhabitants.
- The incidence rate of diagnosed pulmonary TB is on average 100/100,000 inhabitants per annum.
- Of the diagnosed TB cases, on average 50% are diagnosed as sputum-smear positive.
- The level of statistical significance is 0.05 (alpha error).
- The power for detecting an observed difference as statistically significant is 0.80 (Beta error)
- The coefficient of variation is 0.25 (for lack of situational data).
- The additional design effect due to the stepped-wedge design DEFF_sw_ (a consequence of the imbalance between the two comparison groups except halfway the observation period) is between 1.5 and 2.0.

The number of units needed was estimated using the formula by *Hayes and Bennett* [[23]](#endnote-24)for a unmatched group-randomized trial with two arms in which the study outcome is based on disease rates [1]:

C = 1 + (z_α/2_+z_β_)^2^[(λ_0_+λ_1_)/y + k^2^(λ_0_^2^+λ_1_^2^)]/(λ_0_-λ_1_)^2^

in which C is the number of clusters (units) in each study arm, z_α/2_ and z_β_ are the standard normal distribution vales, λ_0_ and λ_1_ are the disease rates in the baseline and the intervention arms, respectively, arm, y is the person-years in each unit, and k is the coefficient of variation. The additional design effect due to the stepped-wedge design was taken into account by inflating in this formula the standard normal distribution values of 1.96 for α=0.05 and 0.84 for β = 0.80 by multiplying these by the assumed design effect DEFF_sw_, as proposed by *Moulton et al*.

In our calculations C was fixed at 14, λ_0_ and λ_1_ were set as indicated below and the additional design effect was varied between 1.5 and 2.0, so that the parameter that changed as a result was y. Assuming fixed population sizes this thus resulted in a minimum observation period for each study arm in a classical group-randomized trial with concurrent allocation of the intervention and equal size, which was then multiplied by 2 to arrive at the total observation period for the stepped-wedge design. We repeated this for total observation periods of 24, 20, 16, 12 and 8 months.

The sample size was calculated based on an average rate of diagnosis of 50/100,000 per annum for the baseline period, and a baseline diagnosis rate of 10/100,000 per annum with bacteriological confirmation through culture (i.e. 20% of sputum smear-negative cases), with a total of 60/100,000 per annum. The minimum diagnosis rate that can then be observed as significantly different from this baseline rate would range from 93/100,000 for a duration of 24 months to 95/100,000 for a duration of 8 months assuming DEFF_sw_=1.5, and from 110/100,000 for a for duration of 24 months to 112/100,000 for a duration of 8 months assuming DEFF_sw_=2.0 (figure 2). Varying the average size of the populations served by each unit from 350,000 to 750,000 has very limited effect on these estimates. For a more conservative, “worst case” scenario of a baseline rate of 20/100,000 (i.e. 40% of all smear-negative TB is bacteriologically confirmed in the baseline period), the corresponding figures would range from 109 to 128/100,000, respectively.

Based on an observed increase in the rate of diagnosed bacteriologically confirmed cases irrespective of smear status. Here we assumed for the baseline period an average rate of bacteriologically confirmed diagnosis 50/100,000 per annum for smear-positive TB of, plus the 10/100,000 mentioned above for smear-negative TB, totaling to 60/100,000 per annum. The minimum diagnosis rate that can then be observed as significantly different from this baseline rate would range from 93/100,000 for a duration of 24 months to 95/100,000 for a duration of 8 months assuming DEFF_sw_=1.5, and from 110/100,000 for a for duration of 24 months to 112/100,000 for a duration of 8 months assuming DEFF_sw_=2.0 (figure 3). Also here, varying the average size of the populations served by each unit from 350,000 to 750,000 has very limited effect. For a more conservative, “worst case” scenario of a baseline rate of 20/100,000 (i.e. 40% of all smear-negative TB is bacetriologically confirmed in the baseline period), the corresponding figures would range from 109 to 110/100,000, and from 128 to 130/100,000, respectively (data not shown). These calculations show that for a duration of 8 months (i.e. each unit contributes on average 4 months to the baseline period and 4 months to the intervention period) the study power is definitely sufficient to detect as statistically significant a relevant increase in the rate of diagnosis or notification of bacteriologically confirmed smear-negative TB, i.e. an approximately 2-fold increase depending on DEFF_sw_. We also calculated the sample size for the detection of a significant increase in sputum-smear negative cases. Considering 50/100,000 per annum for smear-positive TB of, plus the 10/100,000 mentioned above for smear-negative TB, totaling to 60/100,000 per annum during the observation phase. The minimal increase to be detected as stastitically significant would be 17/100,000 for 24 months and 18/100,000 for 8 months, assuming a 1.5 DEFF_sw_; and 20 to 22/100,000 assuming a DEFF_sw_ of 2.0.

## 6. Study duration and phasing

Based on the sample size considerations above the total study duration will be 8 months. Ideally the 14 units would move from the baseline to the intervention on a 2-weekly basis, but this is impractical. Therefore, they will change on a monthly basis in pairs. Simulations by Moulton et al for a similar study design showed that the effect of pair-wise stepped implementation on the DEFFsw was minimal.

Introduction of the GeneXpert as a replacement of sputum smear examination, i.e. moving a unit from the baseline to the intervention group, will be scheduled for a fixed date; the change will be implemented overnight. In the months prior to this date the necessary preparations for the change will be made, including installation of the GeneXpert equipment, delivery of test kits, training of staff, dry run of the assay, and instructions of the staff in the clinics that will request smear examinations.

Below is a graphic representation of the study timeline (by month).

|  | 1 | 2 | 3 | 4 | 5 | 6 | 7 | 8 | 9 | 10 | 11 | 12 | 13 |
| --- | --- | --- | --- | --- | --- | --- | --- | --- | --- | --- | --- | --- | --- |
| Ethical approval | x |  |  |  |  |  |  |  |  |  |  |  |  |
| Visit of all sub sites | x |  |  |  |  |  |  |  |  |  |  |  |  |
| SOP elaboration | x | x |  |  |  |  |  |  |  |  |  |  |  |
| Machine importation |  | x |  |  |  |  |  |  |  |  |  |  |  |
| Staff training |  |  | x |  |  |  |  |  |  |  |  |  |  |
| Observation period |  |  |  | x | x | x | x |  |  |  |  |  |  |
| Linkage of databases and extraction of data |  |  |  |  |  |  |  | x | x | x |  |  |  |
| Data analysis |  |  |  |  |  |  |  |  |  | x | x |  |  |
| Report and scientific paper writing |  |  |  |  |  |  |  |  |  |  |  | x | x |

## 7. In- and exclusion criteria

Since this study aims to assess the impact of introduction of a new diagnostic tool in routine practice, the no in- and exclusion criteria will be introduced in addition to the routine in- and exclusion criteria for diagnosis of TB in the Brazilian health system. According to the current NTP guidelines, every respiratory symptomatic (cough for more than 3 weeks) should be submitted to sputum examination. Likewise, all TB suspect (fever, weight loss, suggestive chest radiograph) will be submitted to examination of spontaneous or induced sputum or bronchoalveolar lavage. This examination will be AFB smear during the observation period and Xpert during the intervention period.

## 8. Data collection

The main study will make use of routinely collected data using two nationwide implemented reporting systems:

1. The TB reporting system. This is part of the nationwide notification system of infectious diseases, SINAN. In this system, notification of TB is mandatory.[[24]](#footnote-2)
2. The laboratory reporting system. The Brazilian Ministry of Health is currently implementing a nationwide laboratory reporting system, GAL. Positive TB testing results of smear examination and culture, as well as of anti-TB drug susceptibility testing, are entered on-line in the system by the diagnosing laboratory. The system will allow for inclusion of positive GeneXpert results.

Both systems will be aligned so that records can be linked on at the individual patient level, using *Reclink*.[[25]](#endnote-25) The variables name, mother’s name, age, neighborhood of residence, date of sputum collection, date of beginning of treatment will be used for linkage. Linkage will allow to detect slightly different writings of the same name (such as accents, “h”), and the other variables will be used to confirm the subject. Thereby a comparison of the GAL and the SINAN system will identify cases that have been diagnosed but not notified. Once data have been extracted, all identification fiels will be excluded from the study database, and all information will be treated anonymously.

## 9. Laboratory methods

Smear examinations, cultures and drug susceptibility testing will be done as per routine. The GeneXpert will be introduced as a routine laboratory procedure and performed in accordance with the manufacturer’s instructions (http://www.cepheid.com) and guidelines drawn based on the validation study that is currently ongoing. No additional laboratory testing will be done. No attempts will be made by the study to interfere with routine laboratorial procedures. Likewise, the routine clinical management will be the same, according to the clinician’s judgment, the NTP guidelines and the good clinical practices.

## 10. Data management and analysis

Over the end of the observation period, data from Manaus and Rio will be extracted from SINAN and GAL as described above. The routine procedures to check errors and linkage will be performed by the designated units in the Health Department, as per routine. This procedure includes checking for duplication, suspected incompatibilities, scale errors. Units are then informed. In Rio, Dr. Valeria Saraceni is responsible for this routine. In Manaus, by the municipal TCP manager, Jair dos Santos Pinheiro. Once this procedure and linkage is done, the identification variables will be deleted and the following variables will be retained for analysis:

- From both SINAN and GAL: patient code, age, sex, health district of residence, sputum smear and culture resultsError: Reference source not found
- From SINAN only: clinic of notification, diagnostic classification (smear-positive/smear-negative/extrapulmonary), treatment history (new, previously treated), date of diagnosis, date of treatment initiation, HIV status, other major co-morbidities.
- From GAL only: positive sputum smear examination details (number of positive smears, highest smear grade obtained, date(s), laboratory); positive mycobacterial culture (number of positive cultures, identification, date(s), laboratory); drug susceptibility results (DST done yes/no, results for each drug tested, date(s), laboratory); positive GeneXpert (number of assays done; highest value obtained; rifampicin resistance yes/no/indeterminate, date(s), laboratory).

General data cleaning will be performed, including checks for duplicate entries, suspected mismatches, range errors, and results communicated to the responsible surveillance units for updating.

The patient code will be assigned for the analysis only and have no reference to patient identifiers.

The data analysis will be performed using STATA and will include:

- 1. Calculation of the number of diagnosed, bacteriologically confirmed pulmonary TB cases by smear status, for each unit, separately for baseline and intervention period.
  2. Calculation of the number of notified pulmonary TB cases, irrespective of bacteriological confirmation, by smear status and in various strata of age, sex and HIV status, for each unit, separately for baseline and intervention period.
  3. Calculation of the number of notified, bacteriologically confirmed pulmonary TB cases by smear status and in various strata of age, sex and HIV status, for each unit, separately for baseline and intervention period.
  4. Calculation of rates for each of these three, by dividing the numbers summed over all units and strata by the summed population denominators, separately for baseline and intervention period, with their 95% confidence intervals, and calculation of the rate ratios and rate differences comparing the two periods with appropriate significance testing.
  5. Multivariate analysis of the observed rate ratios using Poisson regression to adjust for potential confounding due to unbalanced randomization by age, sex, history of TB treatment, HIV status (as far as available), unit and month of enrollment.
  6. Similar calculation of numbers, rates, rate ratios and rate differences by HIV status (as far as available).
  7. Calculation of proportion of diagnosed pulmonary TB patients who are ultimately diagnosed as having MDR-TB, by smear status at initial diagnosis, for each unit, separately for baseline and intervention period.
  8. Calculation of the risk ratios and risk differences for these proportions comparing the two periods with appropriate significance testing.
  9. Multivariate analysis of the observed risk ratios using logistic or log-linear regression (as appropriate, depending on observed proportions) to adjust for potential confounding due to unbalanced randomization by age, sex, history of TB treatment, HIV status (as far as available), unit and month of enrollment.
  10. Calculation, for patients ultimately identified with MDR-TB, of the time interval between diagnosis of pulmonary TB and detection of rifampicin resistance, by smear status at initial diagnosis, for each unit, separately for baseline and intervention period.
  11. Calculation, for patients ultimately identified with MDR-TB, of the time interval between diagnosis of pulmonary TB and initiation of second-line treatment, by smear status at initial diagnosis, for each unit, separately for baseline and intervention period.
  12. Calculation of differences for these time intervals comparing the two periods with appropriate significance testing.
  13. Multivariate analysis of the observed differences in these time intervals using linear to adjust for potential confounding due to unbalanced randomization by age, sex, history of TB treatment, HIV status (as far as available), unit and month of enrollment.

Details of data management will be specified in SOPs. Details of data analyses will be specified in a data analysis plan.

## 11. Sources of bias

The following sources of bias are recognized:

*1. Hospitals in Rio de Janeiro are not included.*

Several referral hospitals in Rio de Janeiro which treat patients with co-morbidities and complex/severe TB cases, do smear examination and other TB diagnostics in their own laboratories, and are thereby outside the Municipal laboratory network. They diagnosed 22% of all TB cases in Rio in 2008, of which 5% are emergency hospitals.

This could affect our results in three ways.

- It would limit the external validity (generalizability) of our findings in that the study results would potentially not apply to clinically more severe cases if the hospitals would preferentially diagnose TB patients with more severe clinical presentations. This scenario seems plausible, thus restricting external validity to severe cases.
- It would affect our sample size calculations if the number of smear-negative cases diagnosed in hospitals was to be disproportionally high, since then the expected number of smear-negative TB diagnoses in the study would be overestimated.
- Selection bias may be introduced (i.e. affecting the study’s internal validity) if referral patterns change during the study. This may happen if patients, who initially are referred to hospitals by family clinics for further diagnosis are no longer referred once the GeneXpert is available in the Municipal lab network. Conversely, hospitals may themselves procure and implement the GeneXpert, potentially resulting in a referral shift in opposite direction. Both scenarios are not expected to play an important role.

*2. In Manaus the “units” are heterogeneous in size and characteristics of the patient populations served.*

Of the three units that will introduce the GeneXpert as part of the roll-out project in Manaus, Policlínica Cardoso Fontes (PCF) in 2009 performed 71% of all smear examinations done in the municipality (14,449 of 20,289) and 72% of positive smear (1149 of 1607). Thereby the PCF unit strongly dominates the phased implementation design. Moreover, its patients come from all over the city and it is difficult to assign a population denominator to the number of diagnoses made; assuming 50/100,000 smear-positive notification rate and 2 smears per smear-positive patient, the population de facto served by PCF is around 1.15M. Consequently, both other units in Manaus, FMTAM and DISA Leste, have relatively few patients.

An additional problem in Manaus is that FMTAM specifically serves HIV-infected patients and diagnoses the majority of co-infected TB patients (the HIV prevalence among TB patients in Manaus being around 19%).

Therefore it was decided that the laboratory at Policlinica Cardoso Fontes (PCF) will have 2 geneXpert machines, because their number of daily smears would be surpassed by one machine capacity (around 32 tests/day, considering their working hours), and will be randomized in balance with Helio Pellegrino in Rio. FMTAM will be randomized with Hospital Raphael de Paula Souza’s laboratory because of the high prevalence of HIV-infected patients in this hospital.

## 12. Quality assurance

*Collection of diagnostic data*

Since the main study attempts to observe routine practice, quality assurance of diagnostic requests, laboratory testing, reporting of results to physicians and recording of notification and laboratory data will be primarily through intensified routine supervision by the responsible TB control and laboratory programs. This intensified supervision will take place already during the baseline period in order to make sure that differences in e.g. quality of recording between baseline and intervention period do not bias the study outcomes. This will be reinforced by brief refresher training in routine diagnosis, reporting and recording of all participating labs and good laboratorial practices. Problems encountered and suggestions for improvement will be laid down in standardized supervision reports (see SOP list below).

*Routine data*

Over the study period, routine quality checks of the SINAN and GAL systems for the two municipalities will be intensified. This will include data cleaning, checks for missing data, and database linking errors. Problems encountered and suggestions for improvement will be laid down in standardized supervision reports (see SOP list below).

*Quality monitoring meetings*

The PIs and study coordinators will have monthly meetings (face-to-face, or by telephone/skype) to discuss progress of the study and quality assurance reports, and take decisions about changes needed in order to upkeep study progress and quality. In additions, ad hoc meetings can be held to take decisions on urgent problems.

# **IV. ETHICAL ISSUES**

For the present study, routine practice will be observed, routine recording and reporting data only will be used, and data that can identify individual patients will not be involved. Only SINAN and GAL data will be used, no clinical or clinical record data will be used. The study will evaluate a procedure that will be carried out as a routine one, as a pilot study for the NTCP for the incorporation of the new diagnostic tool for TB control in the country. Randomization will not be performed by patient, it will instead be done by laboratory. Data analysis will also be performed by groups, not by individual. The new test was already approved by ANVISA[[26]](#footnote-3) (attached) and is already available in private laboratories.

Moreover, there is published evidence that the GX hás a superior accuracy than sputum smear. Além disto, há evidências publicadas na literatura internacional15^-161718^ de que o GX é superior em acurácia à baciloscopia. Although the study aims to evaluate an intervention, this intervention (the introduction of a new diagnostic tool) will be implemented as a routine, programmatic intervention at the group level only. The diagnostic tool concerned will have been cleared by authorities for use in Brazil and adopted by the National TB Control Program. Therefore we request a waiver for individual informed consent, since there are no research subjects, instead, groups of subjects will be routinely submitted to a laboratorial procedure with evidence of better accuracy than conventional tests. Thus, subjects from whom samples will be submitted to the new test will not be exposed to risks, on the contrary, we hope to protect them from using unnecessary hepatotoxic drugs and to detect earlier resistant forms of the disease, avoiding transmission of resistant TB in the community. The principle of non-malfeasance will be respected.

Because this is an intervention in health care units under the responsibility of the involved municipalities, na informed consent Will be presented to the coordinators of the activities and include information on the objectives, methods and expected results of the study.

# **V. RESEARCH TEAM**

Researchers

| **Name** | **Function** | **Link to CV** |
| --- | --- | --- |
| Draurio Barreira | Coordinator | CV attached |
| Betina Durovni | Principal Investigator | http://lattes.cnpq.br/5926314252826641 |
| Reynaldo Dietze | Co- Principal Investigator | http://lattes.cnpq.br/1727805739693861 |
| Solange Cesar Cavalcante | Colaborator | http://lattes.cnpq.br/8833072812952248 |
| Anete Trajman | Colaborator | http://lattes.cnpq.br/7932066626042090 |

Administrative support

| **Name** | **Function** |
| --- | --- |
| Alexandre Menezes | Consultant |
| Anete Trajman | Consultant FAP-project manager |
| Renata da Silva Antunes | Project Assistant |

Advisory Board

| **Name** | **Institution** |
| --- | --- |
| NTP Representatives  Draurio Barreira  Patricia Cafrune | NTP Coordinator, President of the Board  NTP Scientific Department |
| Alexandre Menezes | Bill & Melinda Gates Foundation |
| Marcia Ferreira Teixeira Pinto | Fernades Filgueiras Insitute /FIOCRUZ |
| Valeria Cavalcanti Rolla | Evandro Chagas Research Insitute /FIOCRUZ |
| Joel Keravec | Management Sciences for Health (MSH) |
| Afranio Kritski | TB Research Network and Federal University of RJ |
| Margareth Dalcolmo | Professor Helio Fraga Reference Center/FIOCRUZ |
| Ezio Tavora | Civil Society |

# **VI. LIST OF STANDARD OPERATING PROCEDURES AND FORMS**

The following standard operating procedures (SOPs) and forms will be completed during the preparation phase of the study or used as per routine:

| ***No.*** | ***Product*** | ***Type*** | ***Preparations*** |
| --- | --- | --- | --- |
| **1** | Sputum examination request form | Routine | To be adapted to accommodate GeneXpert |
| **2** | Sputum transport SOP | Routine | Exists |
| **3** | Sputum smear examination SOP | Routine | Exists |
| **4** | Sputum smear reporting form (to clinic) | Routine | Exists |
| **5** | GeneXpert lab SOP | Routine | To be developed before pilot |
| **6** | GeneXpert reporting form (to clinic) | Routine | To be developed before pilot |
| **7** | SINAN recording SOP | Routine | Exists |
| **8** | GAL recording SOP | Routine | To be adapted to accommodate GeneXpert |
| **9** | SINAN-GAL data validation SOP | Routine | To be developed before study |
| **10** | SINAN-GAL data extraction SOP | Specific | To be developed before study |
| **11** | Notification data cleaning SOP | Specific | To be developed before study |
| **12** | Notification data analysis plan | Specific | To be developed before study |
| **13** | Lab supervision checklist | Specific | To be adapted to intensified supervision |
| **14** | Lab supervision report form | Specific | To be adapted to intensified supervision |
| **15** | Clinic supervision checklist | Specific | To be adapted to intensified supervision |
| **16** | Clinic supervision report form | Specific | To be adapted to intensified supervision |

# **IX. BUDGET (in US$)**

|  | Year 1 | Year 2 | Year 3 |
| --- | --- | --- | --- |
| Equipments (GeneXpert) | 245,601.00 | - | - |
| Salaries/RH (investigators, coordinators, supervisors, data mangers) | 142,519.93 | 442,222.58 | 157,185.16 |
| Supplies (cartridges) | 162,000.00 | 648,000.00 | - |
| Services (software license) | 7,062.00 | - | - |
| Travel | - | 6,400.00 | - |
| Total | 557,182.93 | 1,096,622.58 | 157,185.16 |

The total budget (US$ 1,810,990.68) was approved by Bill & Melinda Gates Foundation and the budget for Year 1 is already available, at the rate of US$1=R$ 1.66.

# **X. REFERENCES**

1. . World Health Organization. Facts about tuberculosis. Acesso em 14 Fev 2011. Disponível em <http://www.who.int/features/factfiles/tb_facts/en/index4.html> [↑](#endnote-ref-2)
2. . World Health Organization. Global tuberculosis Control: WHO report 2010. ISBN 978 92 1564069

   Geneva, 218p. Acesso em 14 Fev 2011. Disponível em <http://whqlibdoc.who.int/publications/2010/9789241564069_eng.pdf> [↑](#endnote-ref-3)
3. . World Health Organization. The Global Plan to Stop TB 2011-2015. Transforming the fight towards the elimination of tuberculosis. ISBN 978 92 4 150034 0. Acesso em 14 Fev 2011. Disponível em < http://www.stoptb.org/assets/documents/global/plan/TB_GlobalPlanToStopTB2011-2015.pdf> [↑](#endnote-ref-4)
4. . Ministério da Saúde. Programa Nacional de Controle da Tuberculose. Apresentação PNCT. Acesso em 14 Fev 2011. Disponível em <<http://portal.saude.gov.br/portal/arquivos/pdf/apres_padrao_25_01_11_site.pdf>> [↑](#endnote-ref-5)
5. . Ministério da Saúde. Secretaria de Vigilância em Saúde. Programa Nacional de Controle da Tuberculose. Manual de Recomendações para o Controle da Tuberculose no Brasil. Acesso em 14 Fev 2011. Disponível em <http://portal.saude.gov.br/portal/arquivos/pdf/manual_de_recomendacoes_controle_tb_novo.pdf> [↑](#endnote-ref-6)
6. . Sousa Ludmilla Monfort Oliveira, Pinheiro Rejane Sobrino. Óbitos e internações por tuberculose não notificados no município do Rio de Janeiro. Rev. Saúde Pública. 2011; 45(1): 31-9. Acesso em 14 Fev 2011. Disponível em <http://www.scielo.br/scielo.php?script=sci_arttext&pid=S0034-89102011000100004&lng=en [↑](#endnote-ref-7)
7. . Belo MT, Luiz RR, Hanson C, Selig L, Teixeira EG, Chalfoun T, Trajman A. Tuberculosis and gender in a priority city in the state of Rio de Janeiro, Brazil. J Bras Pneumol. 2010;36(5):621-5. [↑](#endnote-ref-8)
8. . Moure R, Muñoz L, Torres M, Santin M, Martín R, Alcaide F. Rapid Detection of *Mycobacterium tuberculosis* complex and Rifampin Resistance in Smear-negative Clinical Samples using an Integrated Real Time PCR Method. J Clin Microbiol. 2010 Dec 29. [Epub ahead of print] [↑](#endnote-ref-9)
9. . Marlowe EM, Novak Weekley SM, Cumpio J, Sharp SE, Momeny MA, Babst A, Carlson JS, Kawamura M, Pandori M. Evaluation of the Cepheid Xpert MTB/RIF assay for the Direct Detection of *Mycobacterium tuberculosis* Complex from Respiratory Specimens. J Clin Microbiol. 2011 Feb 2. [Epub ahead of print] [↑](#endnote-ref-10)
10. . Filippo S, Mitchelmore I, Pillai P, Mulla R. Rapid diagnosis of *Mycobacterium tuberculosis*using Cepheid XpertTMMTB/RIF PCR. HPA conference, 2010 [↑](#endnote-ref-11)
11. . Bodmer T, Ströhle A. Diagnosing pulmonary tuberculosis in a low prevalence setting: the Xpert® MTB/RIF test. ESM, 2010 [↑](#endnote-ref-12)
12. . Malbruny B, LE Marrec G, Courageux K, Leclercq R, Cattoir V. Rapid and Efficient Detection of *Mycobacterium tuberculosis* by the Cepheid Xpert MTB/RIF Assay, ICAAC, Boston, Set 2010 [↑](#endnote-ref-13)
13. . M.T. Tórtola, L. Nieto, M.G. Codina, N. Martín-Casabona. Detection of *M.tuberculosis* and rifampicin-resistance using a commercial PCR real time technique in respiratory and extrapulmonary samples . [↑](#endnote-ref-14)
14. . Nataša Fajfar, Urška Bidovec-Stojkovič, Manca Žolnir-Dovč. Evaluation of GeneXpert MTB/RIF assay for detection of *Mycobacterium tuberculosis* and rifampicin resistance in a routine laboratory setting in Slovenia. 31st Annual ESM Congress, Bled, Slovenia, July 2010. [↑](#endnote-ref-15)
15. . Ioannidis P, Papaventsis D, Nikolaou S, Karabela S, Panagi M, A. Raftopoulou et A et al. Evaluation of GeneXpert MTB/RIF Assay for MTB Detection and Rifampicin resistance in Athens, Greece. 31st Annual ESM Congress, Bled, Slovenia, July 2010. [↑](#endnote-ref-16)
16. . Naidoo S. Evaluation of Xpert MTB/RIF Assay on pulmonary samples in a high throughput routine laboratory. Pathvine Congress, Cape Town, August, 2010. [↑](#endnote-ref-17)
17. . Naidoo S. Evaluation of GeneXpert MTB/RIF Assay on pulmonary and extrapulmonary samples in a high throughput routine laboratory. ECCMID, Vienna, April, 2010. [↑](#endnote-ref-18)
18. . World Health Organization. ROADMAP FOR ROLLING OUT Xpert MTB/RIF FOR RAPID DIAGNOSIS OF TB AND MDR-TB. 2010. [↑](#endnote-ref-19)
19. . [Boehme CC](http://www.ncbi.nlm.nih.gov/pubmed?term="Boehme CC"%5BAuthor%5D), [Nabeta P](http://www.ncbi.nlm.nih.gov/pubmed?term="Nabeta P"%5BAuthor%5D), [Hillemann D](http://www.ncbi.nlm.nih.gov/pubmed?term="Hillemann D"%5BAuthor%5D), [Nicol MP](http://www.ncbi.nlm.nih.gov/pubmed?term="Nicol MP"%5BAuthor%5D), [Shenai S](http://www.ncbi.nlm.nih.gov/pubmed?term="Shenai S"%5BAuthor%5D), [Krapp F](http://www.ncbi.nlm.nih.gov/pubmed?term="Krapp F"%5BAuthor%5D), et al. Rapid molecular detection of tuberculosis and rifampin resistance. [N Engl J Med.](javascript:AL_get(this, 'jour', 'N Engl J Med.');) 2010;363(11):1005-15. [↑](#endnote-ref-20)
20. . 2º Inquérito Nacional de Resistência. Personnal Communication. [↑](#endnote-ref-21)
21. . Secretaria Municipal de Saúde e Defesa Civil do Rio de Janeiro. Acesso em 14 Fev 2011. Disponível em <http://200.141.78.79/dlstatic/10112/123737/DLFE-220674.htm/TB_incidencia_2008.htm>  [↑](#endnote-ref-22)
22. . Moulton LH, Golub JE, Durovni B, Cavalcante SC, Pacheco AG, Saraceni V, et al. Statistical design of THRio: a phased implementation clinic-randomized study of a tuberculosis preventive therapy intervention. Clin Trials 2007;4:190-199. [↑](#endnote-ref-23)
23. . Hayes RJ, Bennett S. Simple sample size calculation for cluster-randomized trials. Int J Epidemiol 1999;28:319-326. [↑](#endnote-ref-24)
24. Since the submission of this protocol, SINAN has included a field for PCR sputum [↑](#footnote-ref-2)
25. . Camargo K R Jr, Coeli C M. Reclink: an application for database linkage implementing the probabilistic record linkage method. Cad Saude 2000; 16: 439–447. [↑](#endnote-ref-25)
26. Regualtoy agency, similar to FDA in the USA [↑](#footnote-ref-3)
